# Phosphodiesterase beta is the master regulator of cAMP signalling during malaria parasite invasion

**DOI:** 10.1371/journal.pbio.3000154

**Published:** 2019-02-22

**Authors:** Christian Flueck, Laura G. Drought, Andrew Jones, Avnish Patel, Abigail J. Perrin, Eloise M. Walker, Stephanie D. Nofal, Ambrosius P. Snijders, Michael J. Blackman, David A. Baker

**Affiliations:** 1 Faculty of Infectious Diseases, London School of Hygiene & Tropical Medicine, London, United Kingdom; 2 Protein Analysis and Proteomics Laboratory, the Francis Crick Institute, London, United Kingdom; 3 Malaria Biochemistry Laboratory, the Francis Crick Institute, London, United Kingdom; Albert Einstein College of Medicine, UNITED STATES

## Abstract

Cyclic nucleotide signalling is a major regulator of malaria parasite differentiation. Phosphodiesterase (PDE) enzymes are known to control cyclic GMP (cGMP) levels in the parasite, but the mechanisms by which cyclic AMP (cAMP) is regulated remain enigmatic. Here, we demonstrate that *Plasmodium falciparum* phosphodiesterase β (PDEβ) hydrolyses both cAMP and cGMP and is essential for blood stage viability. Conditional gene disruption causes a profound reduction in invasion of erythrocytes and rapid death of those merozoites that invade. We show that this dual phenotype results from elevated cAMP levels and hyperactivation of the cAMP-dependent protein kinase (PKA). Phosphoproteomic analysis of PDEβ-null parasites reveals a >2-fold increase in phosphorylation at over 200 phosphosites, more than half of which conform to a PKA substrate consensus sequence. We conclude that PDEβ plays a critical role in governing correct temporal activation of PKA required for erythrocyte invasion, whilst suppressing untimely PKA activation during early intra-erythrocytic development.

## Abstract 2

Phosphodiesterase β (PDEβ) is essential for blood stage development of the malaria parasite. In its absence, phosphorylation of over 200 proteins is adversely affected, revealing PDEβ to be a central regulator that could be targeted with new antimalarial drugs.

## Author summary

Cyclic nucleotide signalling pathways are ubiquitous in eukaryotes and regulate a plethora of cellular processes. Pathway components include cyclases and phosphodiesterases that synthesise and break down the intracellular second messengers cyclic AMP (cAMP) and cyclic GMP (cGMP); the signal is translated into a cellular response by effector kinases activated by elevated cyclic nucleotide levels. Malaria parasites deploy cyclic nucleotide signalling to regulate virtually every stage of their complex life cycle. Using a conditional gene knockout approach, we investigate the function of phosphodiesterase β (PDEβ) in the disease-causing blood stage parasites. PDEβ disruption causes a severe reduction in erythrocyte invasion and rapid post-invasion death. Although we show that PDEβ can hydrolyse cAMP and cGMP, both parts of the phenotype are linked to elevated cAMP levels and hyperactivation of PKA. Quantitative phosphoproteomic analysis identified sites that are differentially phosphorylated in the PDEβ knockout, revealing a role for cAMP signalling in cellular processes ranging from chromatin organisation to protein synthesis, as well as the regulation of parasite-specific components of the erythrocyte invasion machinery. In summary, PDEβ disruption causes a profound dysregulation of key events during blood stage replication that could be exploited for the development of new antimalarial drugs.

## Introduction

The malaria parasite life cycle comprises extended phases in both a human host and a mosquito vector, but little is known of the control mechanisms that orchestrate progression of parasite development and transmission. Asexually replicating blood stage forms cause all the symptoms and pathology associated with malaria, whereas sexual stage parasites called gametocytes are required to mediate transmission to mosquitoes. Cyclic nucleotide signalling is important at most of the key stages of the parasite life cycle in both the host and vector. A role for cyclic GMP (cGMP)–dependent protein kinase (PKG) has been demonstrated in blood stage egress [1, 2] and invasion [3], gametogenesis [4], ookinete motility [5, 6], and sporozoite motility required for invasion of mosquito vector salivary glands and host hepatocytes [7–9]. Available evidence suggests a role for cyclic AMP (cAMP)–dependent protein kinase (PKA) in blood stage invasion [10–12], cell cycle progression [13, 14], anion conductance, and gametocyte deformability [15, 16] as well as regulated exocytosis of sporozoite apical organelles and hepatocyte infectivity [17]. Two recent studies on cAMP signalling in *Toxoplasma gondii* have shown that absence of one of the three PKA catalytic subunits (PKAc1) leads to premature egress of tachyzoites [18, 19]. These studies also revealed roles for PKAc1 in cross talk with cGMP signalling at this stage of the life cycle. An earlier study established a role for PKAc3 in negative regulation of bradyzoite differentiation [20]. Additional key players in cyclic nucleotide signalling are purine nucleotide cyclases, which synthesise cAMP and cGMP from adenosine triphosphate (ATP) and guanosine triphosphate (GTP), respectively, and cyclic nucleotide phosphodiesterases (PDEs), which break down these messenger molecules by hydrolysis. Cyclic nucleotide levels in the cell are balanced by the opposing action of these two enzyme classes and, upon reaching a concentration threshold, activate their respective cyclic nucleotide-dependent protein kinases, PKA and PKG.

The *P*. *falciparum* genome encodes four PDEs (PlasmoDB identifiers: PDEα, PF3D7_1209500; PDEβ, PF3D7_1321500; PDEγ, PF3D7_1321600; and PDEδ, PF3D7_1470500). Reverse genetic approaches have demonstrated that PDEα, PDEγ, and PDEδ are all associated with cGMP hydrolysis but are not essential for blood stage replication [6, 9, 21–23]. In contrast, previous attempts to delete *PDEβ* in *P*. *falciparum* were unsuccessful, suggesting that the enzyme might be essential for asexual blood stage development. Consistent with this, the *P*. *berghei* PlasmoGEM global gene knockout project and a recent *P*. *falciparum* global transposon mutagenesis project defined *P*. *berghei PDEβ* (*PbPDEβ*,PBANKA_141980) and *P*. *falciparum PDEβ* (*PfPDEβ*) as likely essential based on an extremely low relative growth rate of gene knockout parasites (http://plasmogem.sanger.ac.uk/) [24] and the absence of transposon insertion [25], respectively. Collectively, these data suggest that PDEβ is the only essential PDE in the clinically relevant asexual blood stages of the parasite life cycle. Attempts to express recombinant PDEβ have also been unsuccessful. As a result, its substrate specificity and molecular function in the parasite are unknown.

Here, we have used a conditional genetic approach to investigate the essentiality and role of PDEβ in *P*. *falciparum* blood stage development. This has revealed a critical role in blood stage growth that is likely the result of dysregulated PKA activity.

## Results and discussion

### PfPDEβ is a dual-specific PDE that translocates from a likely apical location to a peripheral membrane of merozoites

PfPDEβ is expressed during asexual blood stage development with mRNA levels increasing in the second half of the approximately 48-hour cycle and peaking in mature schizonts (http://plasmodb.org/). The presence of six putative transmembrane domains distinguishes PfPDEβ from all but one (hPDE3) of the 11 human PDE families that are otherwise soluble [26]. It is currently not possible to predict the substrate specificity of a PDE from its sequence, as this is thought to be defined by multiple components of the binding pocket [27], but sequence comparisons of the catalytic domain of PfPDEβ with selected mammalian PDEs show that 14 of the 15 residues that are invariant amongst all human PDEs are conserved in PfPDEβ (S1 Fig). This suggests that it is a bona fide enzymatically active PDE.

**Fig 1:**
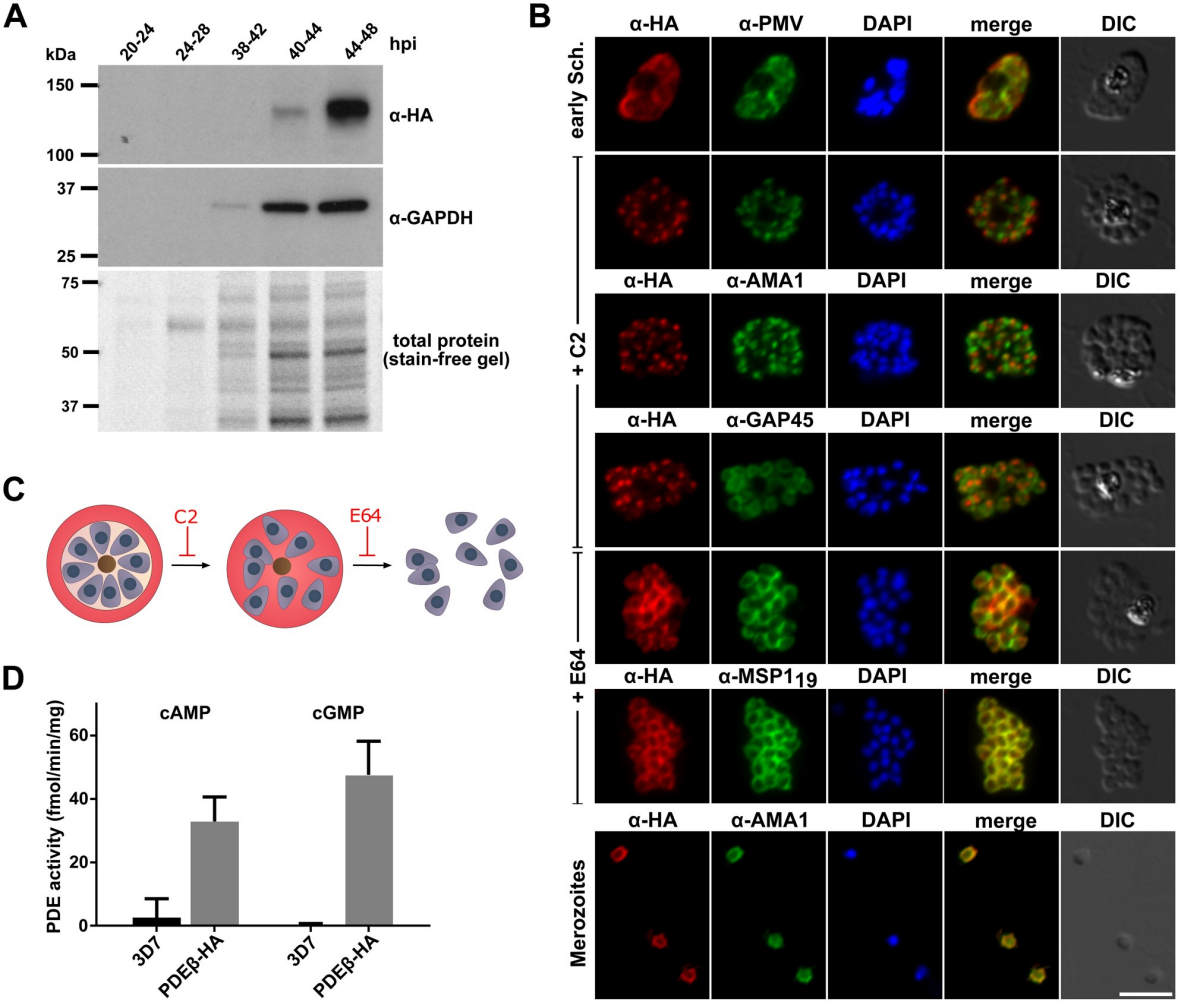
PfPDEβ is a dual-specific PDE that translocates from an apical location to the merozoite plasma membrane. (A) Western blot showing a time course of PfPDEβ-HA expression in the *Plasmodium falciparum* blood stage cycle. Parasites were harvested at the indicated hours post invasion (hpi). Equal parasite numbers were loaded in each lane. Positions of molecular weight markers are indicated. (B) Colocalisation in schizonts by IFA (following inhibitor treatment, left) of PfPDEβ-HA (red) with markers of specific subcellular compartments (green): plasmepsin V (PMV; ER marker), AMA1 (microneme marker), GAP45 (IMC marker), and MSP1_19_ (plasma membrane marker). Nuclear material was visualised by DAPI staining (blue). Merged red and green channels are shown (merge) and a DIC microscopy image is shown to the right. Scale bar, 5 μm. (C) Schematic showing the effects of Compound 2 and E64 on merozoite egress. (D) Hydrolytic activity (fmol product/minute/mg substrate) of PfPDE-HA affinity-purified from parasite extracts, using either cAMP or cGMP as substrate. Activity of control pull-downs from similarly prepared extracts of wild-type *P*. *falciparum* (3D7) are also shown. Data are means from three biological replicates, each performed in triplicate. Error bars, SEM. AMA1, apical membrane antigen-1; cAMP, cyclic AMP; cGMP, cyclic GMP; C2, Compound 2; DIC, differential interference contrast; ER, endoplasmic reticulum; GAPDH, glyceraldehyde 3-phosphate dehydrogenase; GAP45, glideosome-associated protein 45; HA, haemagglutinin; hpi, hours post invasion; IFA, immunofluorescence assay; IMC, inner membrane complex; MSP1_19_, merozoite surface protein 1_19_; PDE, phosphodiesterase; PfPDEβ, *Plasmodium falciparum* phosphodiesterase beta; PMV, plasmepsin V.

Using a transgenic *P*. *falciparum* line in which PfPDEβ was tagged with a triple haemagglutinin (3×HA) tag (PfPDEβ_HA_, S2A and S2B Fig), expression was detectable by immunofluorescence (IFA) throughout blood stage development (S2C Fig). In a western blot time course, a band at around the expected size of the tagged protein (about 136 kDa) was most intense at the late schizont stage (Fig 1A). Full-length PfPDEβ protein was also detected in early and late ring stages (S2D Fig). Further IFA experiments showed that in early schizonts, PDEβ colocalises with the endoplasmic reticulum (ER)–resident protein plasmepsin V (Fig 1B). We then used the PKG inhibitor Compound 2 and the cysteine protease inhibitor E64 in parallel to examine the localisation by IFA of PDEβ at two stages of late schizont development. Compound 2 blocks development of fully segmented schizonts with all the surrounding membranes intact. E64 blocks schizont development at a slightly later stage when the parasitophorous vacuole membrane (PVM) has ruptured (Fig 1C). This approach revealed a dual localisation for PDEβ consistent with a distinct apical signal predominant in Compound 2–arrested schizonts and a pattern reminiscent of a plasma membrane or inner membrane complex (IMC) localisation in E64-arrested schizonts and in free merozoites (Fig 1B). Both localisation patterns were also observed in unblocked schizonts (S2E Fig). These data, combined with the prediction that PDEβ is an integral membrane protein, suggest that the PDEβ is transported via the ER to an apical location (presumably a secretory apical organelle) and then subsequently discharged to the plasma membrane of individual merozoites within mature schizonts.

Previously reported work using recombinant PfPDEα [22, 23] and gene knockout studies on PfPDEδ [21] as well as *P*. *berghei* PDEδ [6] and *P*. *yoelii* PDEγ [9] detected only cGMP hydrolytic activity associated with these three isoforms, with no evidence attributing cAMP hydrolytic activity to any of these malaria parasite PDEs. To establish whether PDEβ is capable of hydrolysing cAMP, we immunoprecipitated the protein from PfPDEβ_HA_ schizont extracts via its epitope tag, followed by a PDE activity assay. This clearly demonstrates that PDEβ is a dual-specific PDE that is able to hydrolyse both cAMP and cGMP in vitro (Fig 1D). We conclude that PDEβ is likely the only blood stage PDE with cAMP-hydrolysing activity.

### PDEβ is essential for asexual blood stage parasite viability

**Fig 2:**
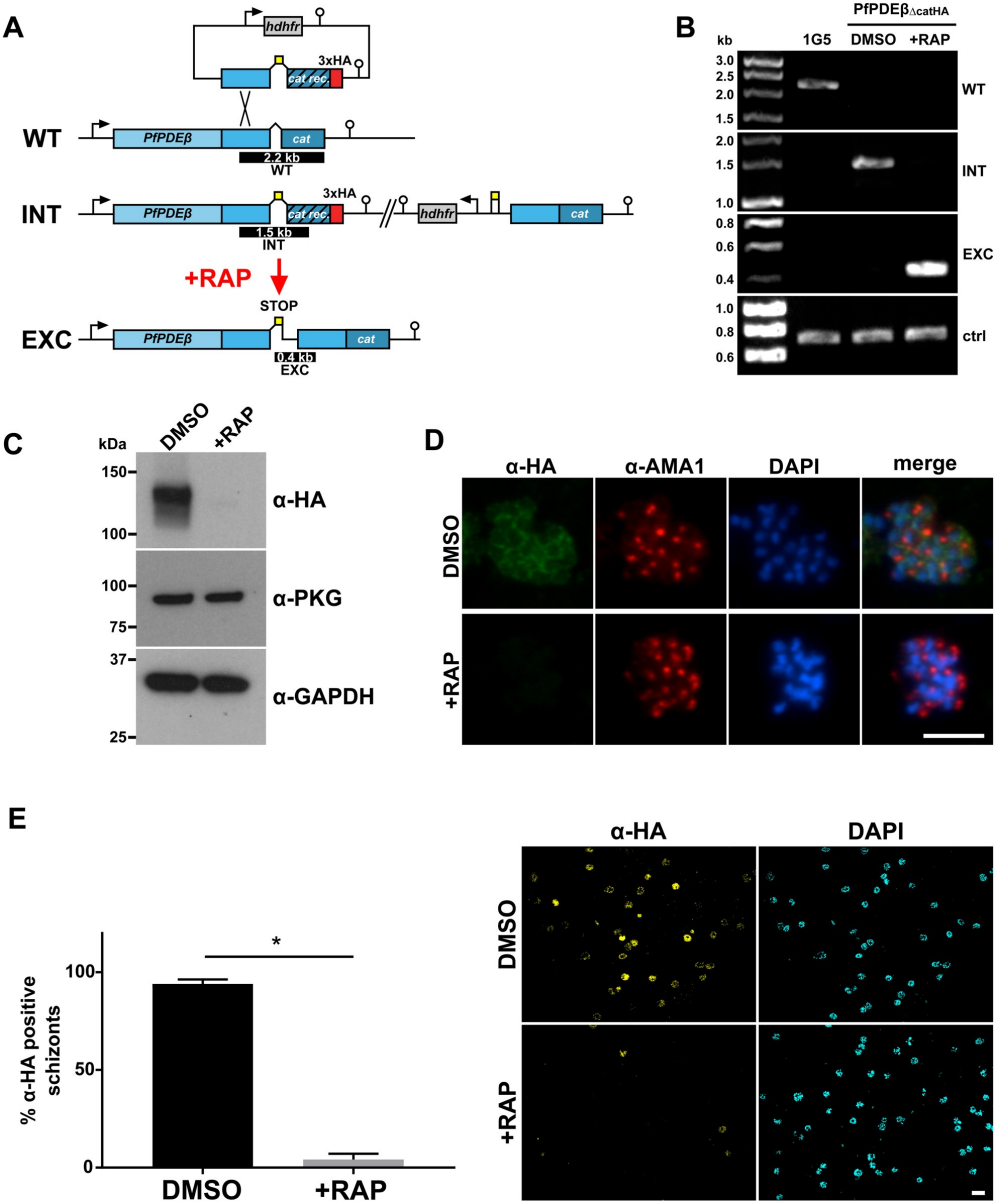
Rapid and efficient conditional disruption of PfPDEβ expression. (A) Schematic of the strategy used for generating a conditional PfPDEβ knockout line (PfPDEβ_ΔcatHA_) by incorporating loxP sites (yellow boxes) flanking the recodonised catalytic domain (hatched box), a triple HA tag (red), and a *hdhfr* selection cassette (grey) into the *PfPDEβ* locus of the DiCre-expressing *Plasmodium falciparum* line 1G5DC. Black arrows denote promoters and lollipops represent transcription terminators. The relative positions are shown of PCR amplicons (black bars) used to confirm the absence of wild-type locus (WT), or correct plasmid integration (INT; introduced by single crossover homologous recombination) into the *PfPDEβ* locus, or RAP-induced excision (EXC) of the floxed sequence. (B) Diagnostic PCR analysis of the *PfPDEβ* locus in the parental 1G5DC line and the PfPDEβ_ΔcatHA_ line before and following RAP treatment. Amplification of an irrelevant target sequence used as a DNA quality control (ctrl). (C) Western blot showing efficient ablation of PfPDEβ-HA protein expression in the schizont stage of the erythrocytic cycle (cycle 0), in which the parasites were RAP treated. Antibodies to PKG and GAPDH were used to control for equal loading. (D) IFA showing the effects of RAP treatment on PfPDEβ-HA protein expression. Cycle 0 schizonts were co-stained with anti-HA (green), anti-AMA1 (red), and DAPI (blue). Scale bar, 5 μm. (E) The efficiency of PfPDEβ_ΔcatHA_ disruption following RAP treatment was determined at the protein level by quantification of anti-HA positive schizonts. Data presented are means from three independent excision experiments. Error bars, 1 SD. More than 100 schizonts were counted per experiment and condition. *, significant by paired *t* test (*p*-value = 0.0002). Representative microscopy images taken at 40× magnification are shown to the right. Anti-HA (yellow), and DAPI (cyan). Scale bar, 10 μm. AMA1, apical membrane antigen-1; ctrl, control; DiCre, dimerisable Cre recombinase; Exc, excision; GAPDH, glyceraldehyde 3-phosphate dehydrogenase; HA, haemagglutinin; IFA, immunofluorescence assay; INT, integration; PfPDEβ, *Plasmodium falciparum* phosphodiesterase β; PKG, cGMP-dependent protein kinase; RAP, rapamycin; WT, wild-type.

To examine the function and essentiality of PDEβ, we used a conditional system employing a rapamycin (RAP)–inducible, dimerisable Cre recombinase (DiCre [28]) to disrupt the *PfPDEβ* gene. We first modified the gene by homologous recombination to introduce *loxP* sites flanking exons 7 to 9, encoding the catalytic domain of the enzyme to produce the conditional knockout line PfPDEβ_ΔcatHA_. PCR analysis of PfPDEβ_ΔcatHA_ confirmed the desired gene modification and also demonstrated RAP-induced excision of the floxed *PfPDEβ* sequence (Fig 2A and 2B). Ablation of expression of the haemagglutinin (HA)–tagged PDEβ catalytic domain following RAP treatment of synchronous ring-stage cultures was confirmed at the protein level by western blot (Fig 2C) and IFA (Fig 2D). Quantification of anti-HA positive schizonts was used to determine the excision rate, which was 95.5% (±3%) (Fig 2E).

**Fig 3:**
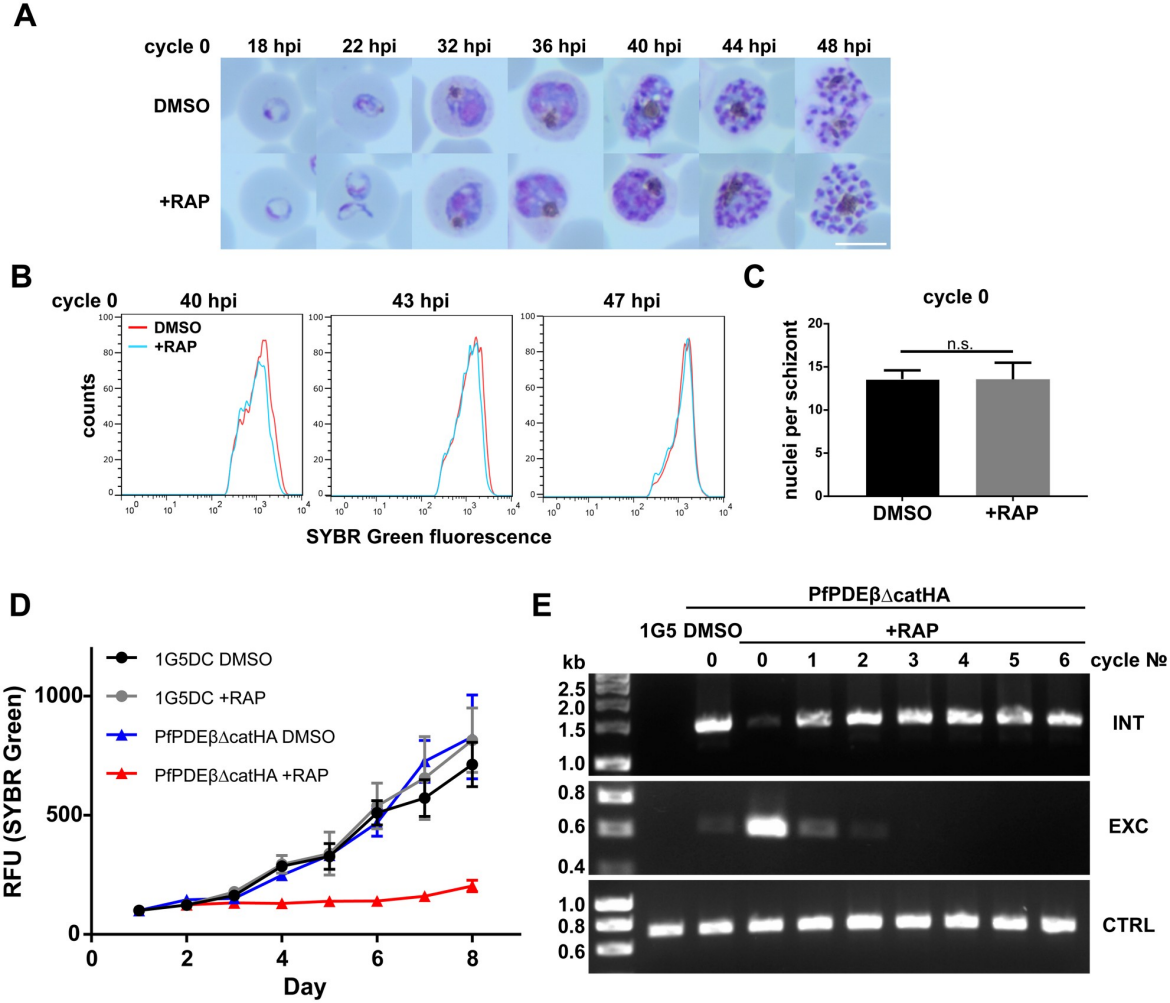
PDEβ disruption has no impact on parasite development in the excision cycle but is lethal to long-term growth. (A) Images of Giemsa-stained blood films showing the morphology of RAP-treated (+RAP) and mock-treated (DMSO) PfPDEβ_ΔcatHA_ parasites at different points in the excision cycle (cycle 0). Highly synchronised parasite cultures were treated whilst at ring stage. Scale bar, 5 μm. (B) DNA content analysis by flow cytometry of cycle 0 PfPDEβ-null schizonts (red plots) compared with untreated schizonts (blue plots). Data representative of three experiments, each performed in triplicate, are shown for each of the three time points. (C) Mean number of PfPDEβ_ΔcatHA_ merozoites per schizont in cycle 0 following mock or RAP treatment. Data were derived from five independent experiments; a total of 100 schizonts were examined on DAPI-stained blood films. Error bars, 1 SD. (D) Growth curves of the PfPDEβ_ΔcatHA_ and 1G5DC parental lines following mock or RAP treatment, derived by measuring parasite DNA content using SYBR Green fluorescence. Each data point is a mean value derived from two biological replicate experiments, each performed in triplicate. Error bars, 1 SD. (E) Diagnostic PCR analysis of the *PfPDEβ* locus in mock and RAP-treated PfPDEβ_ΔcatHA_ cultures over six erythrocytic cycles of parasite replication, showing rapid loss of the signal diagnostic of the excised (PfPDEβ-null) parasites (EXC). Primers used were as described in Fig 2a and 2b. CTRL, control; EXC, excised; hpi, hours post invasion; INT, integration; n.s., not significant by unpaired *t* test (*p*-value = 0.9895); PDEβ, phosphodiesterase β; PfPDEβ, *Plasmodium falciparum* phosphodiesterase β; RAP, rapamycin; RFU, relative fluorescence unit.

The morphology of PfPDEβ_ΔcatHA_ parasites prior to treatment with RAP was indistinguishable on microscopic analysis of Giemsa-stained blood films from that of the 1G5DC parental line parasites. Similarly, no discernible differences in morphology were detected between RAP- and mock-treated PfPDEβ_ΔcatHA_ parasites up to and including the fully segmented schizont stage in the excision cycle (cycle 0) (Fig 3A). Consistent with this, there were no detectable differences in the DNA content of schizonts or the numbers of nuclei per schizont (Fig 3B and 3C). Taken together, these data clearly show that truncation of *PfPDEβ* at the ring stage does not affect intracellular parasite development or schizont maturation in cycle 0.

Monitoring of parasite DNA replication and growth (Fig 3D) for more prolonged periods of approximately 8 days (4 erythrocytic cycles) showed a significant reduction in parasite growth in the RAP-treated cultures. PCR analysis of these cultures showed that PfPDEβ-null parasites did not survive and were quickly outgrown by nonexcised parasites (Fig 3E). Furthermore, viable parasites cloned from these RAP-treated cultures never displayed an excised PfPDEβ locus, indicating that these derived from a minor population of nonexcised parasites (S3 Fig). Collectively, these results show that PDEβ plays an essential role in asexual parasite growth.

### Loss of PDEβ expression leads to a dramatic reduction in invasion and rapid post-invasion death

**Fig 4:**
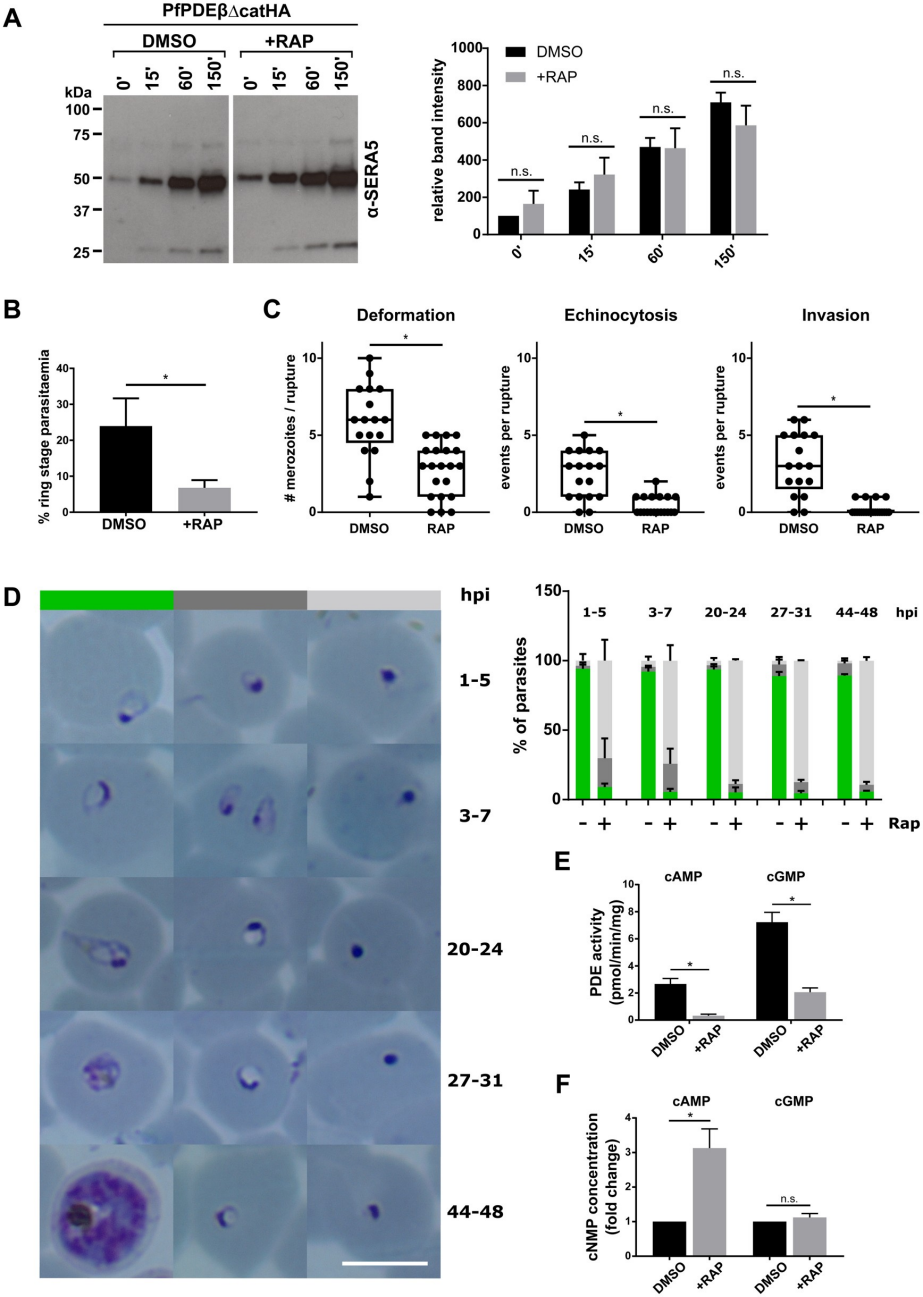
PfPDEβ disruption leads to a severe invasion defect and rapid post-invasion death due to elevated parasite cAMP levels. (A) Western blot analysis of culture supernatants from cycle 0 RAP-treated or DMSO-treated PfPDEβ_ΔcatHA_ schizont cultures over time. Supernatant samples were taken at the indicated times (minutes) following schizont purification. The blot was probed for the soluble PV protein SERA5, a biomarker of merozoite egress. A quantification of band intensities from three independent experiments is shown to the right. Analysis was performed using ImageJ software and values normalised to the DMSO start sample (0'). Means are presented, with error bars representing the standard error of the mean. No significant difference between the two conditions was detected for any time point by *t* test (n.s.). (B) Impact of PfPDEβ disruption on erythrocyte invasion at the transition from cycle 0 to cycle 1. Invasion efficiency was measured using FACS to compare ring stage parasitaemias obtained from DMSO- and RAP-treated PfPDEβ_ΔcatHA_ culture. Data are means from five biological replicates, each performed in triplicate. Error bars, 1 SD. *, significant by paired *t* test (*p*-value < 0.0001). (C) Individual schizont rupture events from RAP (*n* = 21) and mock-treated (DMSO, *n* = 17) PfPDEβ_ΔcatHA_ cultures were followed by video microscopy and scored for the number of merozoites inducing erythrocyte deformation (left), the number of erythrocytes undergoing echinocytosis (middle), and the number of successful invasion events (right). *, significant by unpaired *t* test (*p*-value <0.001). (D) Quantification of parasite morphology in cycle 1, determined by microscopic examination of Giemsa-stained blood films taken at different hpi. Parasites from RAP (+)- and mock (−)-treated PfPDEβ_ΔcatHA_ cultures were counted and scored according to their morphology: normal morphology (green), delayed in development (dark grey), condensed/pyknotic (light grey). Examples of each category and time point are shown in the left panel. Scale bar, 5 μm. Data are means from two independent experiments, independently scored by two researchers. Error bars, 1 SD. At least 300 parasites were scored at each time point and condition. (E) cAMP and cGMP hydrolytic activity (pmol min^−1^ per mg parasite protein) measured in membrane preparations of mature cycle 0 DMSO or RAP-treated PfPDEβ_ΔcatHA_ schizonts. Data are means from five biological replicates, each carried out in triplicate. Error bars, 1 SD. *, significant by Welch-corrected *t* test (*p*-value < 0.0001). (F) Fold changes in cellular levels of cAMP and cGMP in mature cycle 0 DMSO or RAP-treated PfPDEβ_ΔcatHA_ schizonts. Data are from eight biological replicates, each performed in duplicate. Error bars, SEM. *, significant by Welch-corrected *t* test (*p*-value 0.0065); n.s., not significant (*p*-value 0.3155). cAMP, cyclic AMP; cGMP, cyclic GMP; cNMP, cyclic nucleotide monophosphate; FACS, fluorescence-activated cell sorting; hpi, hours post invasion; n.s., not significant; PDE, phosphodiesterase; PfPDEβ, *Plasmodium falciparum* phosphodiesterase β; PV, parasitophorous vacuole; RAP, rapamycin; SERA5, serine repeat antigen 5.

To more precisely define the developmental stage in the erythrocytic cycle at which loss of PDEβ exerted its effect, we next compared egress of mature RAP- or mock-treated PfPDEβ_ΔcatHA_ schizonts at the end of cycle 0. Analysis by western blot of release of the parasitophorous vacuole (PV) protein serine repeat antigen 5 (SERA5) into the culture supernatant (Fig 4A), or flow cytometry (S4A Fig) and time-lapse video microscopy (S1 and S2 Videos; wild type and knockout, respectively), showed that PDEβ deletion had no effect on merozoite egress. We have previously shown that addition of the PDE inhibitor, zaprinast, to mature *P*. *falciparum* schizonts leads to elevated cGMP levels, which activates PKG and triggers merozoite egress, and that this is blocked by addition of a PKG inhibitor (Compound 2 [1]). We have also used this approach previously to show that addition of a PDE inhibitor to mature schizonts triggers elevated cytosolic calcium levels and that, again, this is blocked by PKG inhibition [5]. We used this approach (with zaprinast or 5-Benzyl-3-isopropyl-1*H*-pyrazolo[4,3-*d*]pyrimidin-7(6*H*)-one [BIPPO], a more potent PfPDE inhibitor [29]) to test whether there is any difference in calcium release in PDEβ knockout and wild-type parasites. Levels of cytosolic calcium release were equivalent in PDE inhibitor-treated wild type and PDEβ null schizonts and were PKG dependent (S4C Fig). These results indicate that deletion of PDEβ has no effect on calcium mobilisation, which is required for merozoite egress.

In contrast, flow cytometry of SYBR Green–labelled parasites showed a substantial reduction in invasion efficiency (71% ± 2.78%; *n* = 5) in the RAP-treated PfPDEβ_ΔcatHA_ parasites (Fig 4B and S4B Fig). Merozoites emerging from individual RAP- and mock-treated PfPDEβ_ΔcatHA_ schizonts were followed by video microscopy to assess their competence to induce red cell deformation, echinocytosis, and to conclude successful invasion. Rupture events from RAP-treated schizonts showed a highly significant reduction in all three steps, suggesting that invasion by PDEβ-null merozoites is impaired upstream of tight junction formation (Fig 4C and S3–S7 Videos; the first two are wild type and the last three are knockout). The subpopulation (29%) of PfPDEβ-null parasites that were able to invade consistently gave rise to small, apparently intracellular, merozoite-sized parasites with little or no development of a vacuole or cytoplasm, suggesting a block in development immediately following invasion (Fig 4D). Analysis of the morphology of these dysmorphic intracellular parasites over time revealed that pyknotic parasites were present from the first hour after invasion, whilst some parasites that initially developed a vacuole appeared to rapidly shrink to condensed, dysmorphic forms (Fig 4D and S6A Fig). IFA using two different monoclonal antibodies reactive with distinct proteolytic fragments of merozoite surface protein 1 (MSP1) showed that the majority of newly appearing parasites were intracellular in mock- and RAP-treated cultures (S4D Fig), confirming that a proportion of PfPDEβ-null parasites were able to successfully enter erythrocytes. Together, these results indicate that disruption of PDEβ function leads to approximately a 70% reduction in merozoite invasion and that in the subpopulation of merozoites that successfully invade an erythrocyte, subsequent early post-invasion development is prevented, leading to parasite death prior to ring stage formation.

### PDEβ disruption leads to a dramatic reduction in schizont cAMP and cGMP hydrolytic activity and elevated cellular cAMP levels

To evaluate the impact of PfPDEβ ablation on overall PDE activity in the parasite, schizont extracts were assayed for levels of cAMP and cGMP hydrolytic activity. Cyclic AMP-PDE activity was reduced by approximately 11-fold in extracts of the PfPDEβ-null parasites (Fig 4E), with the small amount of residual cAMP-PDE activity likely being attributed to parasites in which gene excision had not taken place. In contrast, cGMP-PDE activity was reduced by only approximately 3.5-fold, with significant residual activity. These results confirmed that *PfPDEβ* disruption leads to ablation of enzyme activity, and importantly were also consistent with the analysis of immunoprecipitated PDEβ-HA described above in showing that PDEβ is a dual-specific PDE enzyme capable of hydrolysing both cAMP and cGMP. The results also confirmed that there is at least one other PDE expressed in schizonts possessing cGMP-PDE activity, probably PDEα [22]. The results strongly suggest that there is no other asexual blood stage PDE capable of hydrolysing cAMP. In support of this conclusion, whilst PDEβ disruption had no significant effect on cGMP levels in parasite extracts, it resulted in a 3-fold increase in intracellular cAMP levels (Fig 4F). This result provides further confirmation that there is no other PDE capable of regulating cAMP levels in *P*. *falciparum* schizonts, whereas cGMP levels can still be regulated in the absence of PDEβ. The unchanged cGMP levels are consistent with the absence of an egress phenotype in the PDEβ knockout line, as egress is known to be regulated by PKG.

### Phosphoproteome analysis of PfPDEβ-null schizonts reveals hyperactivation of PKA

**Fig 5:**
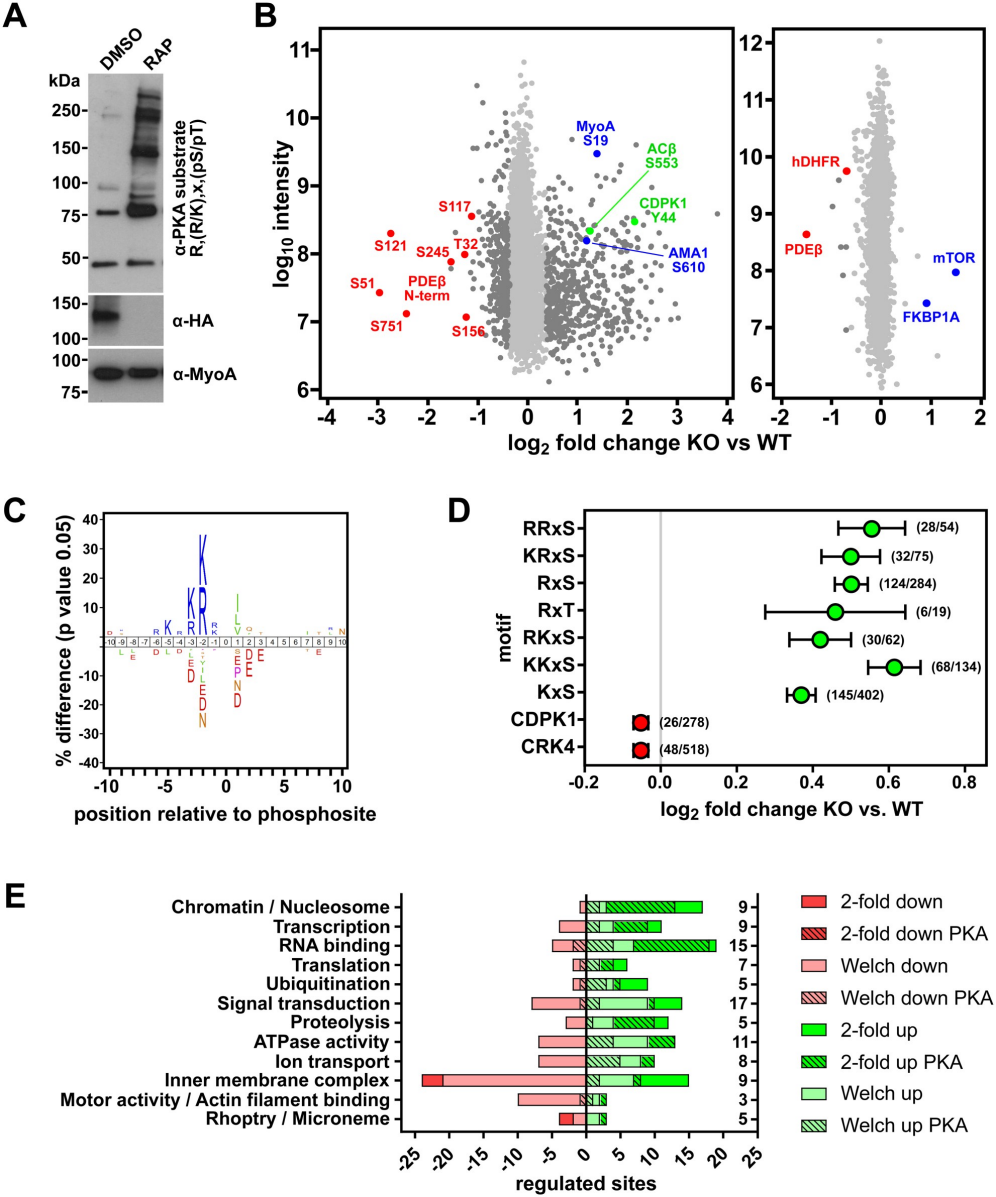
Phosphoproteome analysis shows up-regulation of PKA-mediated phosphorylation in PfPDEβ-null schizonts. (A) Western blot analysis of DMSO- and RAP-treated mature cycle 0 PfPDEβ_ΔcatHA_ schizonts, probed with antibodies specific for the phosphorylated generic consensus PKA substrate motif. The blot was reprobed with an anti-HA antibody to monitor disruption of PfPDEβ-HA expression, as well as an antibody to MyoA as a loading control. (B) Changes in phosphorylation and protein abundance between RAP-treated (PfPDEβ-null, KO) and mock-treated (WT) Compound 2–arrested PfPDEβ_ΔcatHA_ schizonts. Left panel: peptide intensity (log_10_) plotted against log_2_ fold change for 5,374 phosphosites, with significantly altered sites (Welch-corrected *t* test) in dark grey. Seven phosphosites from the PDEβ N-terminal domain (red) and four significantly up-regulated phosphosites in ACβ and CDPK1 (green), as well as MyoA and AMA1 (blue), are highlighted. Right panel: changes in protein abundance, with PfPDEβ, hDHFR, mTOR, and FKBP1A highlighted. (C) Sequence logo showing consensus sequence surrounding phosphosites (position 0) significantly increased in the PfPDEβ-null samples. (D) Motif analysis showing the six motifs most enriched in the PfPDEβ-null samples by 1D annotation analysis (green) and two control kinase motifs (red). CDPK1 and CRK4 motifs used are described in Materials and methods. Data show mean log_2_ fold changes. Error bars, SEM. Numbers in parentheses denote the frequency of the occurrence of the motif in phosphosites significantly up-regulated in the PfPDEβ-null/total number of phosphosites with that motif. (E) Presentation of GO terms dysregulated in PfPDEβ-null schizonts. Bars show numbers of phosphosites up- (green) and down-regulated (red) in the PfPDEβ-null schizonts compared with wild type. Light shades denote sites significantly different by Welch *t* test, and bright colours denote sites >2-fold changed. Hatched bars mark phosphosites with a minimal PKA consensus motif (K/R, x, S/T). The numbers of proteins in each group are indicated on the right. ACβ, adenylyl cyclase β; AMA1, apical membrane antigen-1; CDPK1, calcium-dependent protein kinase 1; CRK4, cdc2-related protein kinase 4; FKBP1A, FK506-binding protein 1A; GO, gene ontology; HA, haemagglutinin; hDHFR, human dihydrofolate reductase; KO, knockout; K/R, lysine/arginine; mTOR, mechanistic target of rapamycin; MyoA, myosin A; PDEβ, phosphodiesterase β; PfPDEβ, *Plasmodium falciparum* phosphodiesterase β; PKA, cAMP-dependent protein kinase; RAP, rapamycin; S/T, serine/threonine; WT, wild type; x, any amino acid; 1D, one-dimensional.

We reasoned that the deleterious effects of *PfPDEβ* disruption on parasite viability might be due to the elevated levels of intracellular cAMP leading to increased phosphorylation of parasite proteins by the parasite PKA. To address this, we first examined extracts of mock- and RAP-treated PfPDEβ_ΔcatHA_ schizonts by western blot with an antibody specific to phosphorylated PKA consensus motifs R, R/K, X, pS/pT (where R is arginine, K is lysine, X is any amino acid and pS or pT denote phosphorylated serine or threonine). PDEβ disruption resulted in an increased number and intensity of antibody-reactive polypeptides, suggesting that the phosphorylation was a result of cAMP-induced ‘hyperactivation’ of PKA following PDEβ disruption (Fig 5A).

To gain insights into candidate proteins underpinning the PfPDEβ-null phenotype and to identify the full complement of putative PKA substrates that become phosphorylated upon *PfPDEβ* disruption, we carried out quantitative mass spectrometric global phosphoproteome analysis of mock- and RAP-treated PfPDEβ_ΔcatHA_ schizonts. Our strategy incorporated PKG inhibition (with Compound 2) to ensure that all the schizonts used for the analysis were synchronised precisely at the point when PKG activity is required for merozoite egress. PKG inhibition also allowed us to distinguish between sites phosphorylated by PKA and PKG, because in other species their consensus substrate sequences are very similar [30]. A total of 5,374 phosphosites were identified, distributed over 1,326 proteins (1,192 *P*. *falciparum* and 134 *Homo sapiens* proteins). Of these, 893 sites were significantly different (Welch unpaired *t* test) between the RAP- and mock-treated samples (Fig 5B left panel and S1 Table), with 341 sites being reduced and 537 sites increased in the knockout. A total of 255 sites were changed by >2-fold, with 239 exhibiting a >2-fold increase but only 16 being decreased by >2-fold in the PDEβ knockout, 7 of which were from the PDEβ N-terminal domain, strongly suggesting that excision of the catalytic domain results in expression of an unstable truncated form of PfPDEβ.

Unphosphorylated peptides present in the phosphopeptide-enriched sample were quantified to show that the vast majority of the 3,170 (2,953 *P*. *falciparum* and 217 *H*. *sapiens*) identified proteins were unchanged in abundance in RAP- and mock-treated PfPDEβ_ΔcatHA_ schizonts (Fig 5B right panel and S2 Table). Only eight proteins were significantly less abundant (Welch unpaired *t* test) in the PfPDEβ-null sample, with PfPDEβ itself (2.7-fold) showing the greatest change. The abundance of human dihydrofoate reductase (hDHFR), used as a drug-resistance selection marker during modification of the *PfPDEβ* locus, was also significantly reduced in RAP-treated samples; this was as expected because the gene is excised together with the *PfPDEβ* catalytic domain upon activation of DiCre (Fig 2A). Two of the few proteins showing a significant increase in abundance in the PfPDEβ-null sample were human proteins, FK506-binding protein 1A (FKBP1A) (1.96-fold) and mechanistic target of rapamycin (mTOR) (2.93-fold). These correspond to the RAP-binding fusion partners used in the DiCre system [28], so this finding is consistent with RAP binding enhancing their stability.

Among the phosphosites significantly increased in the PDEβ knockout, we found a highly significant enrichment in motifs with R or K in the -2 position or -2 and -3 positions relative to the phosphorylation site, resembling mammalian consensus PKA substrate sequences (Fig 5C, S3 Table, and S5 Fig).

Approximately 52% (279/537) of the significantly up-regulated phosphosites and 63% (151/239) of phosphosites increased by >2-fold conform to the minimal PKA consensus motif R/K, x, pS/pT, consistent with hyperactivation of the enzyme in the absence of PfPDEβ (Fig 5B and S5 Fig). It is therefore likely that these data define the *P*. *falciparum* consensus PKA substrate sequence motif (Fig 5C). A 1D rank-based annotation analysis found different variations of K/R, K/R, x, S/T to be the most highly enriched motifs in the PDEβ knockout sample, with RRxS (where R is arginine, x is any amino acid and S is serine) occupying the top rank (Fig 5D and S3 Table). Although the canonical R in position -2 seems to be slightly more enriched, phosphosites with a K in the -2 position, if paired with any basic residue in the -3 position, were also highly enriched. Phosphosites with a K in the -2 position were generally more frequent, which may be a consequence of the A/T-rich *P*. *falciparum* genome. The *P*. *falciparum* PKA may have evolved to better accommodate such K-rich substrates.

Gene ontology (GO) analysis revealed that PfPDEβ disruption led to dysregulated phosphorylation of proteins involved in a range of cellular processes, including chromatin remodelling, transcription, RNA metabolism, translation, and ubiquitination. A number of proteases, ATPases, ion transporters, and signalling components also showed significant changes in phosphorylation. Furthermore, significant changes were found in components of the parasite-specific glideosome as well as rhoptry- and microneme-associated proteins. PKA consensus motifs were overrepresented in the phosphosites up-regulated in the PfPDEβ knockout in these functional groups (Fig 5E and S4 Table). Examination of GO term enrichment, using Gene Ontologizer software, identified significant enrichment of only two GO terms in the significantly down-regulated phosphosites. These were the Biological Process, ‘Ion transport’, and the Cellular Component, ‘Inner membrane pellicle complex’. This reflects a detectable reduction in phosphorylation of significant numbers of proteins within just these two categories in the presence of elevated cAMP levels. One possible explanation for this is that PKA may selectively activate a protein phosphatase to dephosphorylate a restricted number of proteins.

A previous global phosphoproteome of *P*. *falciparum* schizonts showed that 425 of the 2,541 unique phosphosites resembled a consensus PKA sequence, suggesting an important role for this kinase at this life cycle stage [31]. We previously identified 98 *P*. *falciparum* schizont phosphosites that were regulated in a PKG-dependent manner [3]. Interestingly, 46% of these sites were also differentially regulated in the PDEβ knockout (in the presence of a PKG inhibitor), supporting a functional link between the two pathways (S1 Table). Both the *P*. *falciparum* adenylyl cyclase β (PfACβ) and PfPDEβ had a single PKG-dependent phosphorylation site (S_1572_ and S_156_, respectively [3]), which points to a potential mechanism for the regulation of cAMP levels by PKG. Calcium-dependent protein kinase 1 (CDPK1) was identified as a likely direct PKG substrate in the previous study, in which it was phosphorylated at position S_64_. However, in the PDEβ knockout schizonts, an alternative CDPK1 phosphosite (Y_44_) was up-regulated >4-fold (Fig 5B), although which kinase performs this tyrosine phosphorylation event is not known. This also raises the question of whether a second spike of calcium release is required post-egress, as previously implied [32–34].

### The requirement for PKG activity upstream of PKA activation is bypassed in PfPDEβ-null parasites

To seek biological validation of the list of potential PKA substrates, we focused on the phosphosite identified in *P*. *falciparum* myosin A (PF3D7_1342600), MyoA S_19_ (increased by 2.6-fold in the PDEβ knockout). MyoA is a component of the so-called glideosome, a complex of parasite proteins involved in actinomyosin-based motility and host cell invasion [35]. We have previously shown that phosphorylation of MyoA S_19_ in mature wild-type *P*. *falciparum* schizonts is dependent on PKG activity, as treatment with the PKG inhibitor Compound 2 drastically reduces phosphorylation of this residue [3]. However, another study [36] suggested that phosphorylation of MyoA S_19_ is carried out by PKA.

**Fig 6:**
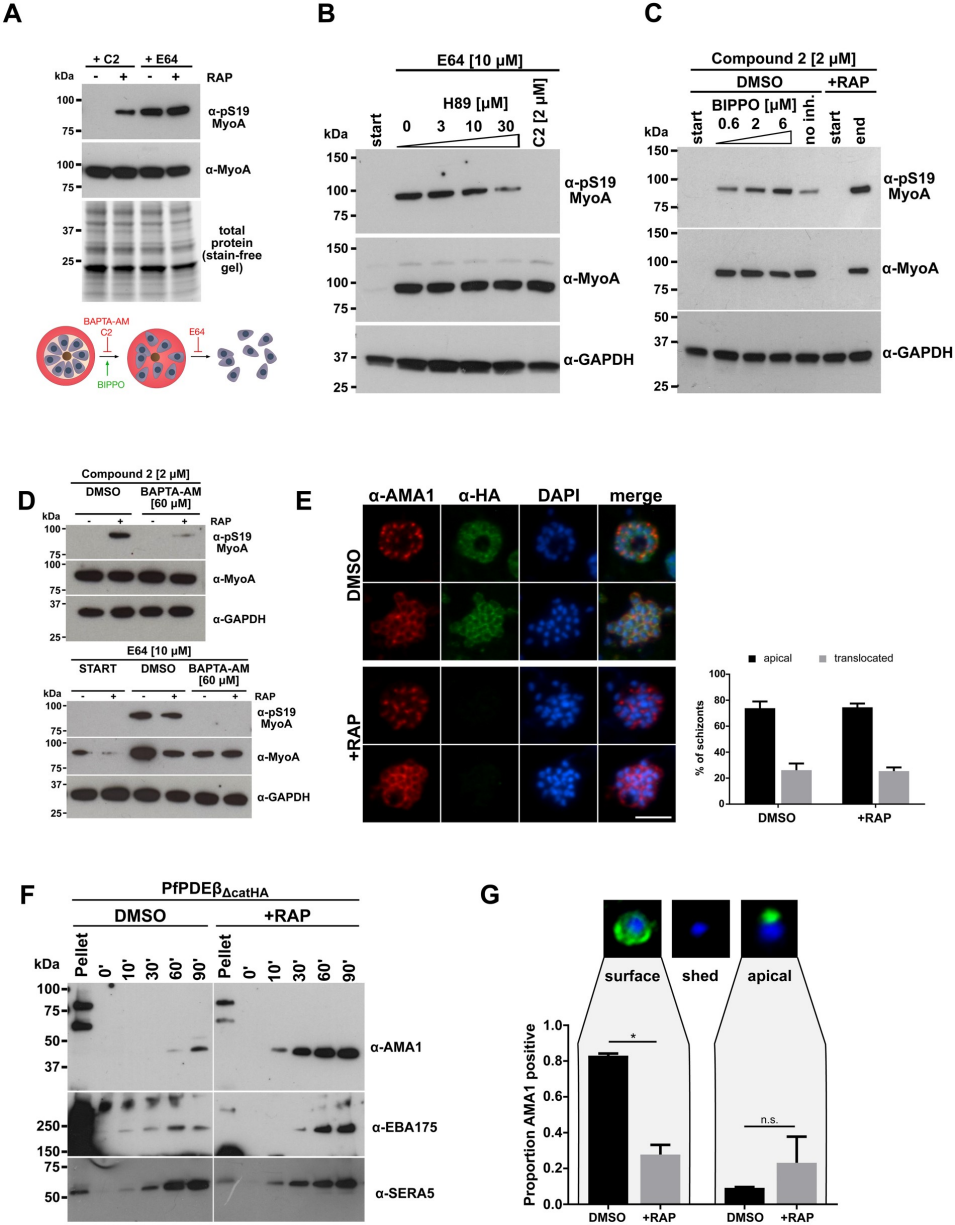
Deletion of PfPDEβ bypasses the need for PKG activity upstream of MyoA S_19_ phosphorylation in mature schizonts and results in premature proteolytic shedding of AMA1. (A) PKG-independent phosphorylation of MyoA in PfPDEβ-null parasites. Western blot of PKG inhibitor–treated (+C2) and E64-treated PfPDEβ_ΔcatHA_ schizont proteins from DMSO- and RAP-treated PfPDEβ_ΔcatHA_ cultures, probed with a phospho-S_19_ MyoA antibody. The blot was reprobed with a polyclonal anti-MyoA antibody to determine the relative amounts of MyoA present in each sample. The lower panel shows total protein levels in the gel prior to blotting. The schematic shows the effect of various compounds on egress: Compound 2 and BAPTA-AM block egress upstream of PVM rupture, BIPPO induces egress, and E64 prevents erythrocyte plasma membrane rupture. (B) The PKA inhibitor H89 inhibits MyoA S_19_ phosphorylation. Western blot analysis of E64-arrested wild-type schizonts (mock-treated PfPDEβ_ΔcatHA_) treated with increasing concentrations of the PKA inhibitor H89. A sample was taken before inhibitor addition (start) and a Compound 2–blocked sample served as negative control. Blots were probed with antibodies against phospho-S_19_ MyoA, total MyoA, and GAPDH as a loading control. (C) The PDE inhibitor BIPPO induces MyoA phosphorylation. Western blot analysis of Compound 2–arrested mock- or RAP-treated PfPDEβ_ΔcatHA_ schizonts, following incubation with increasing concentrations of the PDE inhibitor BIPPO. A sample was taken before inhibitor addition to show the absence of MyoA S_19_ phosphorylation (start). Blots were probed with the same antibodies as in (B). (D) BAPTA-AM inhibits MyoA phosphorylation. Effects of BAPTA-AM on MyoA S_19_ phosphorylation in Compound 2–arrested (top panel) or E64-arrested (bottom panel) PfPDEβ_ΔcatHA_ schizonts following mock or RAP treatment. Western blots were additionally probed with the same antibodies as in B and C. (E) Dual-stained IFAs showing the cellular distribution of AMA1 in RAP- and mock-treated (DMSO) PfPDEβ_ΔcatHA_ schizonts. Schizonts were co-stained with an AMA1 antibody (red) and a HA antibody (green) to detect the presence or absence of PfPDEβ. DAPI (blue) was used to visualise nuclear material. Scale bar, 5 μm. The bar chart (right) shows quantitation of proportions of schizonts displaying apical or peripheral AMA1 staining in E64-arrested PfPDEβ_ΔcatHA_ schizonts. Data presented are from a representative experiment in which >50 schizonts per condition were counted by two researchers. Error bars, 1 SD. (F) Western blot analysis of culture supernatants from RAP- and mock-treated PfPDEβ_ΔcatHA_ purified schizonts sampled over 90 minutes of culture. The blot was probed with antibodies specific for the micronemal proteins AMA1 and EBA175, as well as SERA5, to measure schizont rupture. Positions of molecular weight markers are indicated (left). The blot is representative of at least three independent experiments. (G) Quantitation of AMA1 shedding by IFA in merozoites released from RAP- and mock-treated PfPDEβ_ΔcatHA_ schizonts. Purified merozoites were stained with an antibody against the AMA1 ectodomain. Thumbnails show examples for translocated (surface), shed (−), and micronemal (apical) AMA1 in green. Nuclei were stained with DAPI (blue). The bar charts show the mean proportions of merozoites exhibiting surface (left) and apical (right) AMA1 staining for each condition. More than 100 merozoites were counted by two researchers. Error bars, 1 SD. *, significant by unpaired *t* test (*p*-value = 0.005); n.s., not significant (*p*-value = 0.306). AMA1, apical membrane antigen-1; BAPTA-AM, 1,2-bis(*o*-aminophenoxy)ethane-*N*,*N*,*N′*,*N′*-tetraacetic acid-acetoxymethyl ester; BIPPO, 5-Benzyl-3-isopropyl-1*H*-pyrazolo[4,3-*d*]pyrimidin-7(6*H*)-one; EBA175, erythrocyte-binding antigen 175; GAPDH, glyceraldehyde 3-phosphate dehydrogenase; HA, haemagglutinin; IFA, immunofluorescence assay; MyoA, myosin A; n.s., not significant; PDE, phosphodiesterase; PfPDEβ, *Plasmodium falciparum* phosphodiesterase β; PKA, cAMP-dependent protein kinase; PKG, cGMP-dependent protein kinase; PVM, parasitophorous vacuole membrane; RAP, rapamycin; SERA5, serine repeat antigen 5.

To investigate whether MyoA S_19_ is phosphorylated by PKA or by PKG, we blocked PKG activity (and schizont rupture) in PfPDEβ knockout and control parasites with Compound 2. MyoA S_19_ phosphorylation was not detected in Compound 2–blocked control schizonts, consistent with this being a PKG-dependent event. Surprisingly, however, MyoA S_19_ phosphorylation was abundant in Compound 2–treated PfPDEβ-null schizonts (Fig 6A), strongly arguing for this phosphorylation event being PKG independent in the absence of PfPDEβ. In contrast, MyoA S_19_ phosphorylation accumulated in both control and PfPDEβ-null schizonts incubated with the cysteine protease inhibitor E64, which prevents schizont rupture downstream of PKG activation, confirming that MyoA S_19_ phosphorylation occurs just prior to or at egress (Fig 6A). Levels of MyoA S_19_ phosphorylation in E64-blocked wild-type schizonts were sensitive to the PKA inhibitor H89 in a dose-dependent manner (Fig 6B), consistent with this phosphorylation event being mediated by PKA. In contrast, treatment of Compound 2–blocked wild-type schizonts with the PDE inhibitor BIPPO [29] resulted in concentration-dependent enhancement of MyoA S_19_ phosphorylation, phenocopying the PfPDEβ-null mutant (Fig 6C).

PKG regulates egress upstream of calcium release [1, 37]. To address the sequence of events and relationship of cGMP, cAMP, and calcium signalling with respect to phosphorylation of MyoA, we investigated the effect of the membrane-permeable calcium chelator BAPTA-AM (1,2-bis(*o*-aminophenoxy)ethane-*N*,*N*,*N′*,*N′*-tetraacetic acid-acetoxymethyl ester) on MyoA S_19_ phosphorylation. BAPTA-AM severely reduced MyoA S_19_ phosphorylation in Compound 2–treated PfPDEβ knockout schizonts as well as in E64-blocked wild-type and PfPDEβ knockout schizonts. This indicates that calcium signalling is also required for this phosphorylation event (Fig 6D). It is therefore possible that MyoA_19_ could be phosphorylated by a calcium-dependent protein kinase. Collectively, our results suggest that, although phosphorylation of MyoA S_19_ is PKG dependent, ablation of PDEβ can bypass the need for PKG activity because of the resulting elevated cAMP levels. Further work will be needed to determine whether MyoA S_19_ can be phosphorylated directly by PKA and/or a CDPK in vivo. Interestingly, in the related parasite *T*. *gondii*, MyoA S_21_, which may be functionally equivalent to *P*. *falciparum* MyoA S_19_, has been linked to parasite motility and host cell egress and invasion [38, 39]. Our results therefore suggest that PKG governs cAMP levels as well as PKA activation and that premature phosphorylation of MyoA S_19_ by PKA could lead to premature activation of the actomyosin motor. This may contribute to the severe invasion phenotype observed in PDEβ-null parasites.

### The invasion-related protein AMA1 is shed prematurely from PfPDEβ-null merozoites

A second phosphosite that was significantly up-regulated (2.3-fold) in the PfPDEβ-null schizont phosphoproteome and that had previously been linked to PKA activity is S_610_ of apical membrane antigen-1 (AMA1), a merozoite integral membrane protein that plays an essential role in host cell invasion [40] and that is released onto the merozoite surface from apical secretory organelles called micronemes in a strictly PKG-dependent manner [1]. The S_610_ phosphosite lies within the short cytoplasmic domain of AMA1. Although the exact function of AMA1 phosphorylation remains unknown, it has been shown that the S_610_ modification is one of a series of AMA1 cytoplasmic tail phosphorylation events needed for efficient AMA1 function during invasion [11, 12]. It has also been reported that this event is a prerequisite for subsequent phosphorylation at T_613_ by the parasite glycogen synthase kinase (GSK3) and that both events are required for invasion [12]. In the present study, we did not detect phosphorylation of AMA1 T_613_ in PfPDEβ-null schizonts in which S_610_ phosphorylation is stimulated, although it is possible that the PKG blockade included in our phosphoproteome analysis protocol prevented this subsequent phosphorylation step. Comparative IFA analysis of mock- and RAP-treated PfPDEβ_ΔcatHA_ schizonts showed that AMA1 release remained PKG dependent in PfPDEβ-null schizonts (Compound 2 block) and there was no significant difference in the proportion of parasites exhibiting surface-localised AMA1 in the presence of E64, which blocks the red blood cell membrane rupture step of egress but is permissive for AMA1 release (Fig 6E). However, examination of supernatants from schizont cultures rupturing in the absence of red blood cells detected significantly more shed AMA1 in the PfPDEβ-null samples than in the control, indicating increased levels of AMA1 cleavage (Fig 6F). We also detected increased amounts of shed forms of another micronemal invasion-related protein, erythrocyte-binding antigen 175 (EBA175), in the PfPDEβ-null supernatant sample, whilst there was no difference in levels of the PV protein SERA5 between the two samples, confirming that PfPDEβ disruption does not affect schizont rupture. Confirmation of enhanced AMA1 shedding in the PfPDEβ-null mutant was obtained by IFA analysis of released merozoites, revealing a significant decrease in the proportion of free PfPDEβ-null merozoites reactive with an antibody against the AMA1 ectodomain (Fig 6G). These results suggest that proteolytic cleavage of AMA1 may be increased as a result of enhanced PKA activity in the PfPDEβ-null mutant. Shedding of AMA1 from the merozoite surface is predominantly mediated by the micronemal membrane-bound subtilisin-like protease 2 (SUB2), and to a lesser extent through intramembrane cleavage by the rhomboid-like protease 4 (ROM4) [41–43]. The enhanced shedding of AMA1 may result from enhanced SUB2 activity. Intriguingly, our global phosphoproteomic analysis identified seven phosphosites in ROM4 elevated by greater than 2-fold following RAP treatment, four of which conform with the PKA consensus motif, and two adjacent residues in SUB2 were also significantly changed. It remains to be shown whether any of these phosphorylation events regulate the activity of these proteases.

### Absence of PfPDEβ activity in newly invaded parasites leads to prolonged PKA activity, which interferes with early ring stage development

**Fig 7:**
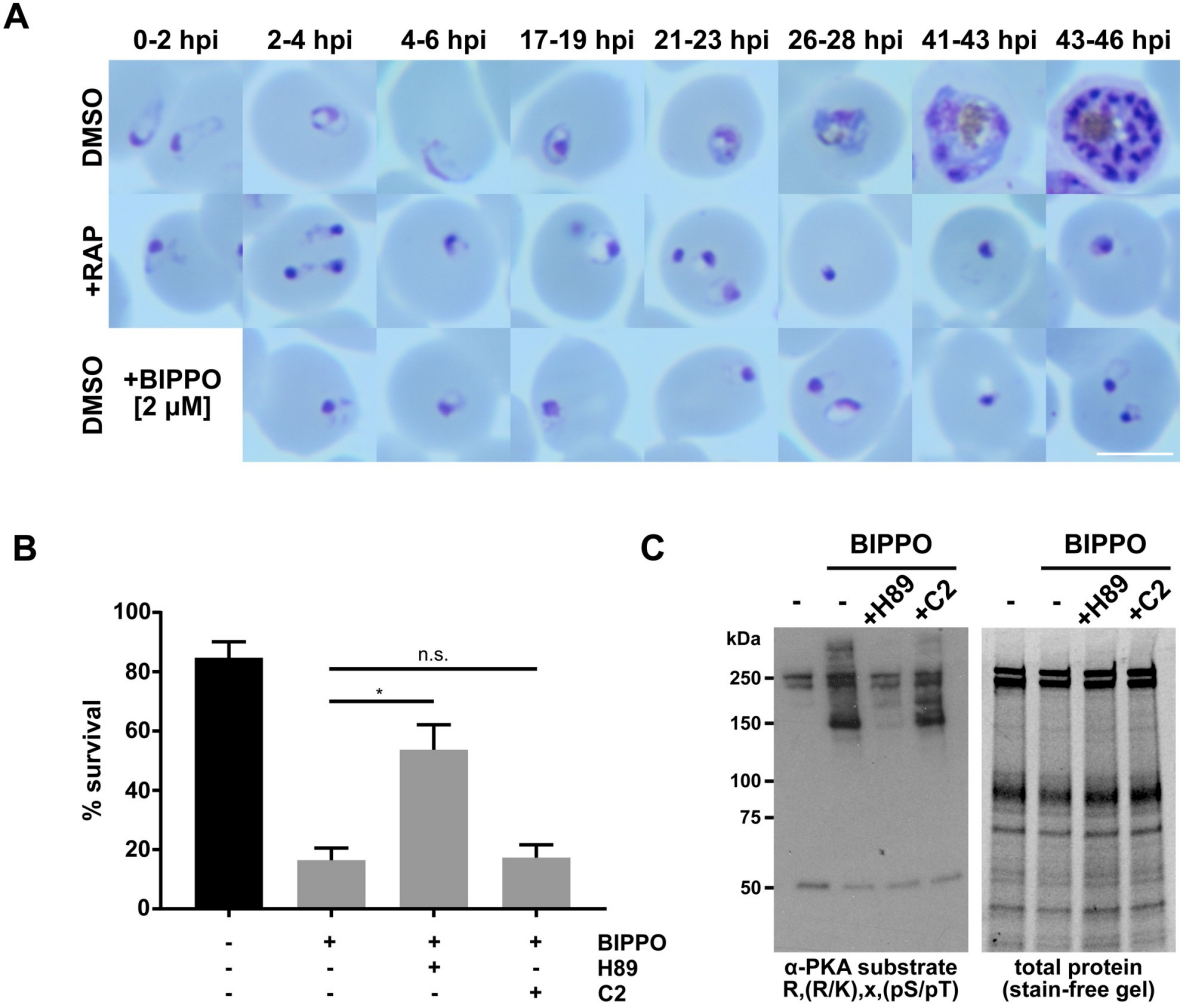
Prolonged PKA activity in newly invaded PfPDEβ-null parasites blocks ring stage development. (A) The PDE inhibitor BIPPO phenocopies the PfPDEβ-null post-invasion phenotype. Giemsa-stained blood films showing the morphology of PfPDEβ_ΔcatHA_ parasites at different time points in cycle 1, following either mock or RAP treatment of the parasites in cycle 0, compared to mock-treated parasites exposed to the PDE inhibitor BIPPO immediately following invasion. (B) The PKA inhibitor H89, but not the PKG inhibitor Compound 2, rescues BIPPO-treated early ring stages. The plots were generated by microscopic analysis of Giemsa-stained smears from ring stage cultures (18–22 hpi) treated with the indicated compounds. Kinase inhibitors (H89 and C2) were added at 1–5 hpi and BIPPO at 2–6 hpi. Scale bar, 5 μm. Data presented are mean counts (evaluated by three independent researchers) from two independent experiments, with >100 parasites counted per condition. Error bars, SEM. *, significant by unpaired *t* test (*p*-value = 0.0005); n.s., not significant (*p*-value = 0.8508). (C) The PDE inhibitor BIPPO induces PKA-dependent phosphorylation in ring stages. Western blot of total protein from ring stages (12–16 hpi) treated for 90 minutes with various inhibitors: BIPPO (2 μM), H89 (30 μM), C2 (2 μM). Lane 1 = no inhibitor control, lane 2 = BIPPO only, lane 3 = BIPPO + H89, lane 4 = BIPPO + C2. The blot was probed with an antibody to phosphorylated PKA substrate motif. The gel was stained for total protein prior to blotting to show equal loading (right panel). BIPPO, 5-Benzyl-3-isopropyl-1*H*-pyrazolo[4,3-*d*]pyrimidin-7(6*H*)-one; hpi, hours post invasion; PDE, phosphodiesterase; PfPDEβ, *Plasmodium falciparum* phosphodiesterase β; PKA, cAMP-dependent protein kinase; pS/pT, phosphoserine or phosphothreonine; RAP, rapamycin; R/K, arginine or lysine.

Activation of PKA at or around egress is thought to be essential for merozoite invasion [10, 11, 31]. Our results show that the requisite increase in cAMP levels is governed by PfPDEβ. However, nothing is known regarding the activity status of PKA following erythrocyte invasion. As described above (Fig 4D), those PfPDEβ-null parasites that successfully invaded underwent immediate developmental arrest, suggesting that PfPDEβ activity is also essential at this stage of the life cycle. To further explore this finding, we examined the effects of treating early ring stage wild-type parasites with the PDE inhibitor BIPPO. As shown in Fig 7A, BIPPO treatment phenocopied the PfPDEβ-null post-invasion phenotype. Previous studies using BIPPO have primarily investigated its effects on cGMP hydrolysis in apicomplexan parasites [18, 29]. Our new results demonstrating that this inhibitor is able to target PDEβ show that it has the potential to affect both cAMP and cGMP hydrolysis in *Plasmodium* blood stages. Reasoning that both the PfPDEβ-null and BIPPO-mediated phenotype are likely caused through elevated cAMP or cGMP levels and inappropriate activation of the respective effector kinases PKA or PKG, we examined whether we could rescue the PfPDEβ knockout and PDE inhibitor phenotype by treating early ring stages (0–2 h post-invasion) with PKG or PKA inhibitors. Survival of PfPDEβ-null parasites was not significantly extended by the addition of either kinase inhibitor (S6C Fig), presumably because they had been exposed to elevated cAMP levels over a long period, causing extensive dysregulation and irreversible damage on multiple levels. In contrast, treatment of wild-type ring stage parasites with the PKA inhibitor H89 partially reversed the effects of BIPPO, resulting in normal ring stage development (Fig 7B and S6B Fig). Treatment with the PKG inhibitor Compound 2 did not reverse BIPPO-mediated killing, clearly attributing the observed phenotype to elevated cAMP levels and untimely PKA activity (Fig 7B). To further link the PfPDEβ-null and PDE inhibitor phenotypes to untimely PKA activation, we treated ring stage parasites with BIPPO alone or in combination with kinase inhibitors. Total parasite lysates analysed by western blot reveal a clear increase in reactivity, with an antibody against phosphorylated PKA substrate motif in the BIPPO-treated sample. Addition of the PKA inhibitor H89 reduced BIPPO-induced antibody reactivity to control levels, whereas the PKG inhibitor Compound 2 did not, confirming that the post-invasion phenotype is likely due to untimely PKA rather than PKG activity (Fig 7C).

Future work will be needed to understand the dynamics of PDEβ function in blood stage malaria parasites, how its translocation from an apical to a peripheral location impacts on the regulation of local cyclic nucleotide levels, and how this might relate to the differential hydrolysis of cGMP and cAMP during merozoite egress and invasion.

In conclusion, we have shown that PfPDEβ is a dual-function enzyme that is the only PDE responsible for regulation of cAMP levels in blood stage malaria parasites. Ablation of PfPDEβ results in hyperactivity of PKA, with the resulting dysregulated phosphorylation leading to either complete loss of merozoite invasive capacity or lethal defects in parasite development immediately post-invasion. We also provide the first direct genetic evidence for PKA dependence of MyoA S_19_ and AMA1 S_610_ phosphorylation in *P*. *falciparum*. Several licensed drugs (e.g., Roflumilast, Sildenafil, and Pentoxifylline) that target human PDEs are widely used to treat a range of disorders [26, 44]. Our results revealing that PfPDEβ is essential for blood stage *P*. *falciparum* replication suggest that PDE inhibitors targeting this enzyme could be developed as new antimalarial drugs, particularly if they also displayed activity against the parasite PDEs expressed in gametocytes and pre-erythrocytic stages of the parasite life cycle.

## Materials and methods

### Small molecules and antibodies

WR99210 was a kind gift from Jacobus Pharmaceuticals (New Jersey), RAP and the cysteine protease inhibitor E64 were purchased from Sigma, the PKG inhibitor Compound 2 was synthesised by MRC Technology (London, United Kingdom), the PKA inhibitor H89 was obtained from TOCRIS Biosciences, the PDE inhibitor BIPPO [29] was a kind gift from Philip E. Thompson (Monash University, Australia). Calcium chelators BAPTA-AM and Fluo-4 AM were purchased from Thermo Fisher Scientific, and the calcium ionophore A23187 was from Sigma-Aldrich.

Rat monoclonal anti-HA tag antibody (clone 3F10) and the same antibody conjugated to agarose beads were purchased from Roche LifeScience. Rabbit anti-AMA1 antibody raised against the ectodomain was described previously [45]. Rabbit anti-EBA175 is described in [43]. Rabbit anti-GAP45 [46] and rat anti-MyoA antisera [3] were kind gifts from Judith Green (The Francis Crick Institute, London, UK). Monoclonal mouse antibodies to MSP1-19 (2F10) and MSP1-83 (89.1) have been described previously [47, 48]. A mouse monoclonal antibody to Plasmepsin V was a kind gift from Daniel E. Goldberg (Washington University School of Medicine in St. Louis, USA). A mouse monoclonal antibody against PfGAPDH was a kind gift from Claudia Daubenberger (SwissTPH, Basel, Switzerland), rabbit anti-SERA5 is described in [49], rabbit anti-PKG antibody was from ENZO life sciences (New York), rabbit anti pS19-MyoA phospho-antibody was raised against the phosphopeptide ‘N’-RRV[pS]NVEAFDKC conjugated to KLH and double purified on the phosphopeptide, followed by passing through its nonphosphorylated counterpart [3]. A rabbit monoclonal antibody specifically reacting with phosphorylated PKA substrate consensus motif (R,K/R,X,pS/pT) was purchased from Cell Signaling Technology.

All fluorescently labelled secondary antibodies used were highly cross-adsorbed and either conjugated to Alexa 488 (green) or Alexa 594 (red) (Molecular Probes).

### Construction of the PfPDEβ-HA tagging and PfPDEβ conditional knockout plasmid

A construct based on the pHH1_SERA5del3DC vector [28] was generated to C-terminally tag the endogenous *Pfpdeβ* locus with a 3×HA tag. The construct contained a 0.9-kb 3′ fragment of the *PfPDEβ* gene, to facilitate single crossover recombination, fused to the HA tag. A loxP site was placed downstream and the 3′UTR of *P*. *berghei* DT. The construct also contained a second loxP site and a hDHFR gene, which confers resistance to the antifolate WR99210. The resulting plasmid pPfPDEβ-HA was originally generated with the intention of conditionally ablating PfPDEβ function by excision of the 3′UTR flanked by loxP sites.

To create the PfPDEβ conditional knockout plasmid, a synthetic, partially recodonised (*Spodoptera frugiperda* codon usage) PfPDEβ sequence was synthesised by GenScript. The sequence comprised 1 kb of native *P*. *falciparum* PfPDEβ targeting sequence containing native PDEβ sequence to drive recombination by single crossover, a loxP site inserted into intron 6 (of 8), and the remaining 819 bp of the PfPDEβ exonic sequence were recodonised to prevent recombination downstream of the loxP site. A triple HA tag was added to the 3′ end of the sequence, followed by a stop codon. The synthetic PDEβ gene containing the internal loxP site and the recodonised sequence was cloned into pPfPDEβ-HA, replacing the 0.9-kb fragment to yield pPfPDEβ_ΔcatHA_.

### *P*. *falciparum* culture and synchronisation

*P*. *falciparum* erythrocytic stages were cultured in human A^+^ erythrocytes (National Blood Transfusion Service, London, United Kingdom) and RPMI 1640 medium (Lifetech) supplemented with 0.5% AlbuMAX type II (Gibco), 50 uM hypoxanthine, and 2 mM L-glutamine according to standard procedures [50]. Tightly synchronised parasites were obtained by purification of segmented schizonts on a 70% Percoll (GE Healthcare) cushion, addition of fresh erythrocytes to allow invasion for 1 to 3 hours shaking, followed by another Percoll purification to remove unruptured schizonts and sorbitol lysis of the pellet to obtain highly pure and synchronous ring stages.

### Transfection of *P*. *falciparum* blood stages

Genetic manipulation of *P*. *falciparum* parasites was carried out as previously described [4]. A total of 80 μg of precipitated plasmid DNA was resuspended in 400 μL cytomix (120 mM KCl, 0.15 mM CaCl_2_, 2 mM EGTA, 5 mM MgCl_2_, 10 mM K_2_HPO_4_/KH_2_PO_4_, and 25 mM HEPES [pH 7]). The DNA-cytomix solution was added to 250 μL of the packed ring stage cultures at 5% to 10% parasitaemia and the sample electroporated at 950 μF capacitance and a voltage of 0.31 kV using a GenePulser Xcell (Bio-Rad). Twenty-four hours later, the selection drug WR99210 was added at 5 nM concentration. Once parasites were visible, the cultures were cycled off the drug for 3 weeks, then on the drug until parasites reached 1% parasitaemia for one to four cycles.

### Limiting dilution to generate clonal parasite lines

Ring stage cultures were counted using a haemocytometer and diluted to give 0.25 parasites per well in a 96-well plate at 2% haematocrit. Culture media was replaced every 3 days. After 14–21 days, positive wells were identified using a lactate dehydrogenase assay [51].

### Integration and excision PCRs

Integration of the HA-tagging plasmid pPfPDEβ-HA into the *Pfpdeβ* (PF3D7_1321500) locus was verified using primers Int F 5′ GTTGAAAAGCAGTACAATAATGTTCCTTATC 3′ and Int R 5′ CGGGATCATAAACCTCGATTG 3′, and the following primers were used to detect the WT locus and the absence of integration: WT R 5′ GCCAAGTCGAATGGAAAGATATTG 3′ and WT F 5′ GTTGAAAAGCAGTACAATAATGTTCCTTATC 3′. Integration of the PDEβ loxP plasmid pPfPDEβ_ΔcatHA_ into the same locus to create PfPDEβ_ΔcatHA_ was confirmed by PCR using primers specific for the integrated locus: int-F 5′ GTTCTTCAAATGGTTGTGTAAAATTAT 3′ and int-R 5′ GGCCAATGTCGTGGCAGATG 3′. Cre recombinase–mediated excision of the PDEβ catalytic domain (exons 7 to 9) was monitored using primers specific for the excised locus: exc-F 5′ GTAATAAGAATGAATAGGCATATATGT 3′ and exc-R 5′ TGAACATTGAAATTTGTATCCGTCT 3′. The 3′ end of the PDEβ coding region unaffected by plasmid integration or Cre recombinase–mediated excision served as DNA quality and loading control: 3′end-F 5′ CAACTAAACCAATGTAATATTTT 3′ and 3′end-R 5′ CGGGATCATAAACCTCGATTG 3′. Primers int-F and 3′end-R were combined to specifically amplify the wild-type locus.

### SYBR Green growth assays

Synchronous ring stage cultures were adjusted to 0.5% parasitaemia, set up in 96-well flat-bottom plates at 1% haematocrit, and triplicate samples frozen down daily for the duration of the assay. Eventually, cells were lysed in 20 mM Tris, 5 mM EDTA, 0.008% saponin, 0.08% Triton X-100, 1× SYBR Green I (Molecular Probes), pH 7.5 [52], and read in a fluorescent plate reader at 485-nm excitation and 535-nm emission. Relative fluorescence units were normalised based on the day 0 sample and plotted. To determine EC_50_ concentrations for inhibitors used in this study, the same assay was conducted with 2-fold serial dilutions of the test compounds in triplicate wells and termination of the assay after 72 hours.

### Immunofluorescence microscopy

Dried blood films were fixed in 4% formaldehyde and permeabilised with 0.1% Triton X-100 in PBS. Blocking and antibody reactions were carried out in 3% bovine serum albumin in PBS and washed with PBS. Slides were air-dried and mounted with ProLong Gold Antifade Mountant containing DAPI (Thermo Fisher Scientific). Images were acquired on a NIKON Eclipse Ti fluorescence microscope fitted with a Hamamatsu C11440 digital camera and overlaid in ICY bioimage analysis software (icy.bioimageanalysis.org). Pure merozoites were obtained by dual MACS (Miltenyi Biotec) purification. Rupturing schizont cultures were isolated on the magnet, put back into culture for 45 minutes, then run through the MACS column again, and the flowthrough containing merozoites was centrifuged at 3,500*g* for 5 minutes. Merozoite preparations were smeared on glass slides, air-dried, and fixed with cold methanol. Blocking and antibody reactions were carried out as described above.

### Time-lapse video microscopy

Segmented schizonts were purified from RAP- or mock-treated PfPDEβ_ΔcatHA_ cultures as described above, introduced into custom-made viewing chambers [28], and imaged on a Nikon Eclipse Ni-E widefield microscope with a Hamamatsu C11440 camera and a Nikon N Plan Apo λ 100x/1.45NA oil immersion objective. For egress videos, images were taken at 5-second intervals over a total of 30 minutes. Individual egress events were cropped, trimmed, and converted to video file format in ICY bioimage analysis software. For invasion videos, images were taken every 150 ms for 8 minutes following schizont rupture and processed using the Nikon NIS elements AR software. Merozoites from each rupture event were followed up and scored for their ability to deform the host cell, induce echinocytosis, and complete invasion.

### Microscopy of Giemsa-stained blood films

Thin blood films were air-dried, methanol fixed, and stained with Giemsa’s azure-eosin-methylene blue (Merck) and imaged on an Olympus BX51 microscope fitted with an Olympus SC30 digital colour camera through a 100× oil immersion objective.

### Quantification of PDEβ KO post-invasion death dynamics

RAP-mediated excision was performed on PfPDEβ_ΔcatHA_ as described above, but treating 3/4 of the whole culture and leaving 1/4 for the control to adjust for the reduced invasion expected in the PDEβ KO. Segmented schizonts from RAP- and DMSO-treated cultures were purified and fresh erythrocytes added to allow invasion for 4 hours and obtain a ring stage parasitaemia of 8% to 10% for both conditions. Giemsa-stained thin blood films taken at five different time points spanning the whole intra-erythrocytic cycle were assessed blind by two different researchers and parasites assigned to either of three morphological categories: normal morphology, delayed, or pyknotic/condensed. More than 300 parasites were scored per time point and condition.

### Inhibitor treatments of early ring stages

Wild-type parasites (3D7) were synchronised to a 2-hour invasion window as described above to obtain a pure culture with 7% to 10% ring stage parasitaemia. Kinase inhibitor treatments were started at 2–4 hours post-invasion, 1 hour before addition of the PDE inhibitor BIPPO. The PKA inhibitor H89 was used at 16.3 μM (approximately 1×EC_50_), the PKG inhibitor Compound 2 at 1.5 μM (approximately 3×EC_50_), and the PDE inhibitor BIPPO at 1.2 μM (approximately 3×EC_50_). Giemsa-stained thin blood films taken at 19–21 hours post-invasion were scored for their viability. More than 100 parasites per condition and experiment were counted blind by three researchers each.

### FACS analysis to measure DNA content, egress, and invasion

Parasite cultures were set up in triplicate wells per condition and fixed with 4% formaldehyde/0.1% glutaraldehyde in PBS containing 1× SYBR Green I (Molecular Probes) for 30 minutes at room temperature. Fixative was washed out with PBS and SYBR Green fluorescence read on a BD LSR II Flow Cytometer (Becton Dickinson). Data were analysed using FlowJo 7 analysis software (Becton Dickinson). For schizont DNA content analysis, the distribution of SYBR Green fluorescence was displayed as a histogram. To analyse schizont rupture and ring stage formation over time, schizontaemia and ring stage parasitaemia were calculated using high or low SYBR Green fluorescence, respectively.

### Parasite protein extraction, SDS PAGE, and immunoblotting

Parasites were released from host erythrocytes by saponin lysis and PBS-washed pellets resuspended in 2–3-pellet volumes of NP-40 lysis buffer (10 mM Tris, 150 mM NaCl, 0.5 mM EDTA, 0.5% NP-40, pH 7.5, + cOmplete protease inhibitors [Roche]), incubated on ice for 10 minutes, and supernatants collected after centrifugation at 15,000*g* for 15 minutes at 4 °C. Reducing SDS sample buffer was added and proteins separated on 4%–15% Mini-PROTEAN TGX Stain-Free Protein Gels (Bio-Rad). Proteins were transferred to nitrocellulose membranes in a Trans-Blot Turbo Transfer System (Bio-Rad) and blocked with 10% skimmed milk in PBS/0.1% Tween-20. Antibody reactions were carried out in 1% skimmed milk in PBS/0.1% Tween-20 and washed in PBS/0.1% Tween-20. Washed membranes were incubated with Clarity Western ECL substrate (Bio-Rad) and exposed to X-ray film.

### Egress assays

Schizonts were Percoll purified from synchronised PfPDEβ_ΔcatHA_ cultures containing mainly segmented schizonts and some young ring stages. Purified schizonts were resuspended in RPMI, 100 uL aliquots distributed in 96-well plates, and assay started immediately. At different time points, culture supernatants were separated from parasite material by centrifugation followed by purification through 0.22-μm Costar Spin-X centrifuge filters (Corning). Presence of SERA5 in culture supernatants was quantified by western blot and used as a measure of schizont rupture. Western blots were probed with anti-AMA1 and anti-EBA175 antibodies to detect differences in adhesin shedding dynamics.

### Calcium release assays

Changes in the levels of intracellular free Ca^2+^ in response to PDE inhibitors were measured in purified late schizonts. Schizonts were resuspended in warm Ringer buffer (122.5 mM NaCl, 5.4 mM KCl, 0.8 mM MgCl_2_, 11 mM HEPES, 10 mM D-Glucose, 1 mM NaH_2_PO_4_, pH 7.4) to 1–2 × 108 parasites/mL (25 μL packed cell volume per 1 mL Ringer buffer). A total of 2 μL of 5 mM Fluo-4 AM (Thermo Fisher) was added per 1 mL of parasite preparation. Cells were incubated in the dark with Fluo-4 AM at 37 °C for 45 minutes. Cells were then washed twice in warm Ringer buffer and incubated for 20 minutes to allow for de-esterification of the AM ester. This was followed by a further two washes. The pellet was resuspended in Ringer buffer at 1–2 × 10^8^ parasites/mL and plated out on a 96-well plate.

Baseline Fluo-4 fluorescence in each well was read at 488-nm excitation and 525 emission using a SPECTRAmax M3 microplate fluorimeter (Molecular Devices) preheated to 37 degrees Celcius at 20-second intervals for a period of 3 minutes. The plate was removed from the reader onto a heat block prewarmed to 37 °C, and cell suspensions were transferred to wells containing test compounds to give the desired final concentrations (ionophore A23187 [20 μM], BIPPO [2 μM], zaprinast [100 μM], and Compound 2 [2 μM]). The plate was placed back in the plate reader and read for a further 5 minutes at 20-second intervals. Relative fluorescence units from individual reads were averaged and averaged baseline reads subtracted. Results are presented as percentage of ionophore control.

### Parasite lysate preparation for enzyme assays

Packed *P*. *falciparum* schizonts were obtained by saponin lysis and resuspended in ice-cold 5 mM Tris-HCl (with EDTA-free protease inhibitors), centrifuged repeatedly at 16,000*g* for 10 minutes at 4 °C, and the supernatant aspirated to remove residual RBC material until the supernatant was clear. The pellet was then resuspended in 250 μL PDE lysis buffer (10 mM Tris-HCl, pH 7.5, 150 mM NaCl, 0.5% Nonidet P-40, and EDTA-free protease inhibitors) per 50 μL of sample, incubated on ice for 30 minutes and centrifuged at 16,000*g* for 20 minutes at 4 °C. Supernatants were added to PDE assay.

### Immunoprecipitation of PDEβ-HA

Pull-downs of the transgenic HA epitope–tagged PDEβ-HA were performed using the anti-HA Affinity Matrix (Roche, 11815016001) that incorporates immobilised rat monoclonal antibody (clone 3F10). Packed parasite pellets were obtained by saponin lysis. The pellet was resuspended in 250 μL ice-cold PDE lysis buffer per 50 μL of sample, incubated on ice for 30 minutes with occasional mixing, and centrifuged as described above. The supernatant was adjusted to 500 μL with PDE dilution buffer (10 mM Tris-HCl, pH 7.5, 150 mM NaCl, and EDTA-free protease inhibitors) to give a final detergent concentration of less than 0.2%, and the pellet discarded. A total of 20 μL of the matrix was washed twice with dilution buffer (centrifuged at 30 seconds 13,000*g* to pellet the matrix) to equilibrate the anti-HA affinity matrix. The lysate sample was then added to the equilibrated anti-HA affinity matrix, and incubated at RT for 2 hours with constant mixing. After incubation, the matrix was pelleted at 13,000*g* and the supernatant removed. The beads were washed twice with ice-cold dilution buffer and were added to the PDE assay.

### PDE assays

PDE activity in *P*. *falciparum* particulate fractions and pull-down assays was measured by a scintillation proximity assay (SPA) using yttrium silicate–based SPA beads (Perkin Elmer, RPNQ0150). Scintillant is incorporated into beads, which bind to the primary phosphate groups of noncyclic 5′AMP or GMP, and the assay relies on the fact that cAMP and cGMP are unable to bind. Assays were carried out in flexible 96-well plates (Perkin Elmer, 1450–401) in a 100-μL volume. A total of 90 μL of protein sample diluted in PDE assay buffer (50 mM Tris-HCl, 8.3 mM MgCl_2_, and 1.7 mM EGTA) was added to each well, and 10 μL of a cNMP dilution (5 μL of [^3^H] cNMP tracer [Perkin Elmer cAMP-NET275250UC, cGMP-NET337250UC] in 995 μL PDE assay buffer) was added to start the reaction. Plates were incubated at 37 °C for 1 hour. Reactions were terminated by addition of 50 μL of resuspended PDE SPA beads (reconstituted to 20 mg/mL in distilled H_2_O). Plates were sealed with Plateseal (Perkin Elmer) briefly shaken and then incubated for 20 minutes at RT to allow the beads to settle. Scintillation was measured using a Wallac 1450 Microbeta Counter (Perkin Elmer) for 30 seconds. An initial dose-response assay was performed with doubling dilutions of the sample to ensure the substrate was not depleted during the course of the assay. The initial sample was diluted to give roughly 30% hydrolysis of the cyclic nucleotide.

### Measurement of intracellular cyclic nucleotide levels

Relative intracellular cAMP and cGMP in mature schizonts were measured using ELISA-based FluoProbes high-sensitivity chemiluminescent assay kits (Interchim). Mature schizonts were Percoll purified from RAP- or DMSO-treated PfPDEβ_ΔcatHA_ cultures followed by saponin lysis and two PBS washes. Parasite pellets were directly lysed in sample diluent for 10 minutes at room temperature, centrifuged at 20,000*g* for 15 minutes, and the supernatant collected and diluted 1:5 in sample diluent. Samples and cyclic nucleotide standards were acetylated according to the manufacturer’s high sensitivity protocol. Standards and samples were run in triplicates on the same plate and luminescence read with a Spectramax M3 plate reader. The standard was fitted to a sigmoidal curve and used to determine cyclic nucleotide concentrations in parasite samples.

### Global phosphoproteomics and proteomics

The PfPDEβ_ΔcatHA_ line was synchronised to a 2-hour invasion window, as described above, to obtain 5×10^8^ ring stages. The culture was split into two, and one half was treated with 100 nM RAP and the other with vehicle (DMSO). RAP and DMSO were washed out 3 hours later. Schizonts were Percoll purified 40 hours post-invasion and grown for a further 8 hours in the presence of 1.5 μM PKG inhibitor Compound 2. Fully segmented schizonts were then harvested and host erythrocytes lysed with 0.15% saponin (Sigma) in the presence of complete protease inhibitors (Roche) and washed twice in PBS plus protease inhibitors, snap-frozen and stored at −80 °C. Parasite proteins were extracted with 10 volumes of 9 M urea in 50 mM HEPES, pH 8.5, containing benzonase (Sigma) at 100 units/mL. Lysates were sonicated with a probe sonicator (three bursts of 15 seconds on ice), centrifuged at 15,000*g* for 30 minutes at 4 °C, and protein content determined by a Bradford protein assay. A total of 1.05 mg of each protein sample was reduced by 5 mM dithiothreitol, alkylated with 10 mM iodoacetamide, and quenched with 7.5 mM dithiothreitol. Samples were diluted with 50 mM HEPES to reduce the urea concentration to <2 M prior to trypsin digestion. Peptides were desalted using a C18 Sep-Pak cartridge under vacuum, each sample divided into three (3 × 350 μg), and dried. Samples were resuspended in 50 mM HEPES and 30% (v/v) acetonitrile, and the corresponding TMTsixplex (0.8 mg) label (resuspended in anhydrous acetonitrile) was added (TMT6-126, -127, -128 to +RAP; TMT6-129, -130, -131 to DMSO). Hydroxylamine was added to quench the reaction, and the samples were mixed and desalted using a C18 Sep-Pak cartridge. Dried peptide mixtures were resuspended in 1 M glycolic acid + 80% acetonitrile + 5% trifluoroacetic acid and added to titanium dioxide beads (5:1 [w/w] beads:protein), washed under acidic pH, and eluted from the beads by adding 1% ammonium hydroxide followed by 5% ammonium hydroxide, and dried by vacuum centrifugation. One third of the material was desalted with the use of a C18 Stage Tip, and the other two thirds was fractionated and desalted by the use of a Pierce High pH Reversed-Phase Peptide Fractionation Kit.

An Orbitrap Fusion Lumos was used for data acquisition. Desalted phosphopeptide mixtures were resuspended in 25 μL 0.1% trifluoroacetic acid and injected twice (10 μL per injection); high-pH fractionated phosphopeptide mixtures were resuspended in 15 μL 0.1% trifluoroacetic acid and injected once (10 μL). Each run consisted of a 3-hour gradient elution (75 μm × 50 cm C18 column), with higher-energy collision dissociation (HCD) being the selected activation method. MaxQuant [53] (version 1.5.2.8) was used for all data processing. The data were searched against UniProt extracted *H*. *sapiens* and *P*. *falciparum* proteome FASTA files. A decoy database containing reverse sequences was used to estimate false discovery rates and the false discovery rate was set at 1%. Default MaxQuant parameters were used with the following adjustments: reporter ion MS2 with the sixplex TMT isobaric labels was selected, Phospho(STY) was added as a variable modification, and ‘Filter labeled amino acids’ was deselected. Protein levels (MaxQuant ProteinGroups) were calculated from nonphosphorylated material quantified in the phosphopeptide-enriched sample. The mass spectrometry proteomics data have been deposited to the ProteomeXchange Consortium via the PRIDE [54] partner repository with the dataset identifier PXD009157. CDPK1 and CRK4 substrate motifs used as controls were combinations of motifs proposed previously [55, 56]. Individual motifs are shown in S5 Fig.

### Data analysis

All graphs were created using GraphPad Prism7 and statistical significance tests performed in the same software.

Statistical significance tests on phosphoproteome data (Welch *t* test with S0 = 0.2, permutation-based FDR set to 0.05 and 250 randomisations), motif analyses, 1D rank-based annotation enrichment [57], scatterplots, and all matrices were created in Perseus 1.4.0.2.

Sequence logo was created using IceLogo (https://iomics.ugent.be/icelogoserver/) using all 5,374 phosphosites (31 amino acid sequence windows) identified in this study as the reference dataset and all phosphosites significantly (Welch *t* test) increased in the PfPDEβ KO sample as the experimental set.

GO enrichment analysis was performed on Gene Ontologizer (http://ontologizer.de/) using the latest ontology and *P*. *falciparum* gene association files downloaded from http://www.geneontology.org. Parent-Child-Union was used as the calculation method and *p*-values adjusted using the Bonferroni correction. All gene IDs present in the phosphoproteome dataset served as reference against gene IDs representing significantly changed phosphosites in the PfPDEβ KO sample.

## Supporting information

S1 Fig Alignment of the catalytic domains of selected human PDEs and the *Plasmodium falciparum* PDEs. A total of 14 of the 15 residues (red) that are invariant amongst all of the 11 human PDE families are conserved in PfPDEβ. Additional residues identical in all the aligned sequences are highlighted in grey. Human PDE catalytic domains representing either dual-specific (HsPDE1, EAW96791), cAMP-specific (HsPDE4, NP_001032418), or cGMP-specific (HsPDE9A, AAC39778) types are aligned with the catalytic domains of the four *P*. *falciparum* PDEs. The 16 helical segments derived from studies on mammalian PDEs are depicted by coloured bars to reflect the three sub-domains: blue, NH2-terminal; orange, middle; and green, COOH-terminal, defined previously [58]. Functional residues defined in mammalian PDEs are indicated by the following: green ‘h’, hydrophobic clamp that binds the purine base; blue ‘m’, metal ion coordinating; red ‘n’, nucleotide recognition; and purple ‘c’, hydrolysis [59]. The amino acid numbers are shown to the right of the sequences, and the total number of amino acids in each protein is shown at the end in brackets. cAMP, cyclic AMP; cGMP, cyclic GMP; COOH, carboxyl; NH2, amino; PDE, phosphodiesterase; PfPDEβ, *Plasmodium falciparum* phosphodiesterase β.
(TIF)

S2 Fig Generation of a PfPDEβ-HA line and tagged protein expression across the intra-erythrocytic cycle. (A) Schematic showing the approach to C-terminally tag the endogenous *PfPDEβ* gene with a 3×HA tag. The plasmid construct was transfected into a line expressing a RAP-inducible Cre recombinase, upon activation of which the 3′ untranslated region (3′UTR) is excised. Excision of the 3′UTR did not result in the anticipated mRNA destabilisation and concomitant knockdown of protein levels. However, the created line proved useful for PDEβ localisation and enzymatic activity studies. Black arrows denote promoters and lollipops represent transcription terminators (grey circle represents the heterologous *P*. *berghei dhfr-ts* terminator). Positions of PCR amplicons verifying integration as well as absence of wild-type locus (see [B]) are indicated by black bars. (B) Diagnostic PCRs showing correct integration (INT) of the plasmid via single crossover into the PDEβ locus as well as absence of wild-type locus (WT) for two clones. The band obtained with primers specific for the plasmid (PLS) shows that multiple plasmid copies are integrated into the target locus. (C) Representative images of formaldehyde-fixed thin smears of ring, trophozoite, and schizont stages of PDEβ-HA parasites probed with rat anti-HA monoclonal antibody (green). Parasite nuclei are stained with DAPI (blue). (D) Full-length PfPDEβ-HA is expressed in early and late ring stages. Total lysates obtained from synchronous, high parasitaemia ring stage cultures were subjected to western blot analysis with monoclonal antibodies to the HA tag and the PfGAPDH. A section of the gel stained for total protein (stain-free gel) is shown as a loading control. (E) Dual-staining IFAs performed on thin smears of unblocked PfPDEβ-HA schizont cultures. Slides were stained with anti-HA (red), EBA175, or AMA1 (green). Nuclear material was visualised by DAPI (blue). Merged red and green channels are shown (merge) and a DIC microscopy image is shown to the right. Scale bar, 5 μm. AMA1, apical membrane antigen-1; DIC, differential interference contrast; EBA175, erythrocyte-binding antigen 175; HA, haemagglutinin; hpi, hours post-invasion; IFA, immunofluorescence assay; INT, integration; PDEβ, phosphodiesterase β; PfGAPDH, *Plasmodium falciparum* glyceraldehyde 3-phosphate dehydrogenase; PfPDEβ, *Plasmodium falciparum* phosphodiesterase β; PLS, plasmid-specific primer; WT, wild-type; 3×HA, triple haemagglutinin.
(TIF)

S3 Fig Analysis of clones obtained from a RAP-treated PfPDEβΔcatHA culture confirms essentiality of PDEβ for blood stage growth. (A) PCR analysis of the PDEβ locus in six clones grown from a RAP-treated culture grown in the absence of WR99210 for 4 weeks. None of the six clones carried the excised PDEβ locus. A weak or absent integration-specific band is consistent with partial or full reversion of the unexcised PDEβ locus to wild type. (B) Growth curves for the six clones determined after 4 weeks of culture in the absence of WR99210 by daily measurements of DNA content via SYBR Green fluorescence (RFU). Means of technical triplicates are presented. Parasite clones were grown in the absence (−WR) and presence (+WR) of WR99210. Drug challenge reveals that three out of six clones had largely reverted to the drug-sensitive wild-type PDEβ locus. PDEβ, phosphodiesterase β; PfPDEβ, *Plasmodium falciparum* phosphodiesterase β; RAP, rapamycin; RFU, relative fluorescence unit; WR, WR99210.
(TIF)

S4 Fig Comparison of egress, invasion, and PKG-dependent calcium release in PDEβ KO and wild-type parasites. (A) Decline in schizontaemia over time in synchronous DMSO- and RAP-treated PfPDEβΔcatHA schizont cultures as determined by FACS on SYBR Green–stained cultures. Samples were taken every 45 minutes (T1–T10) and 12 hours later (T11). The data are mean schizontaemia (starting schizontaemia adjusted to 5%) from two biological replicates carried out in triplicate, and the error bars denote the standard deviation. (B) Increase in ring stage parasitaemia over time in synchronous DMSO- and RAP-treated PfPDEβΔcatHA cultures as determined by FACS on SYBR Green–stained cultures. Samples were taken every 45 minutes (T1–T10) and 12 hours later (T11). The data are mean ring stage parasitaemia (untreated starting parasitaemia adjusted to 1%) from two biological replicates carried out in triplicate, and the error bars denote the standard deviation. (C) DMSO- and +RAP–treated PfPDEβΔcatHA schizonts loaded with the membrane-permeable fluorescent calcium reporter Fluo-4 AM were exposed to PDE inhibitors BIPPO (2 μM) and zaprinast (ZAP) (100 μM), with or without the PKG inhibitor Compound 2 (C2) (2 μM), and calcium release measured as Fluo-4 fluorescence. Inhibitor-induced calcium release was normalised to values obtained for calcium ionophore, which was set to 100%. Data presented are means from three independent experiments performed in technical triplicates. Error bars, SEM. A paired *t* test showed no significant difference in response to either PDE inhibitor between wild-type (DMSO) and PDEβ KO schizonts. (D) IFA analysis suggests that mononucleated PDEβ knockout parasites are inside erythrocytes. Blood films of RAP- and mock-treated PfPDEβΔcatHA cultures containing segmented schizonts and early ring stages were stained with monoclonal antibodies recognising MSP1-83, which is shed at invasion, and MSP1-19, which is carried into the erythrocyte. There was no difference between the two conditions in the proportion of parasites staining with either antibody. Nonsignificant unpaired *t* test *p*-values are as follows: MSP1-83, 0.1368; MSP1-19, 0.5442. BIPPO, 5-Benzyl-3-isopropyl-1*H*-pyrazolo[4,3-*d*]pyrimidin-7(6*H*)-one; C2, Compound 2; FACS, fluorescence-activated cell sorting; IFA, immunofluorescence assay; KO, knockout; MSP, merozoite surface protein; PDE, phosphodiesterase; PDEβ, phosphodiesterase β; PfPDEβ, *Plasmodium falciparum* phosphodiesterase β; PKG, cGMP-dependent protein kinase; RAP, rapamycin; ZAP, zaprinast.
(TIF)

S5 Fig Differential phosphorylation between PDEβ knockout and wild-type schizonts at phosphosites carrying PKA and control motifs. Intensity plots visualising log2 fold changes in phosphorylation at individual PKA and control motifs (in red) between PDEβ KO (+RAP) and wild-type (DMSO) schizonts. The total of 5,374 phosphosites are shown in grey. CRK4 motifs as identified by Ganter and colleagues, 2017 [56], and CDPK1 motifs as identified by Kumar and colleagues, 2017 [55], were unchanged. Motifs are shown above graphs. *n* = total number of phosphosites identified with a particular motif. Numbers in parentheses denote the ratio of phosphosites with a particular motif that were found to be significantly increased (Welch *t* test) in the PDEβ KO sample. CDPK1, calcium-dependent protein kinase 1; CRK4, cdc2-related protein kinase 4; KO, knockout; PDEβ, phosphodiesterase β; PKA, cAMP-dependent protein kinase; RAP, rapamycin.
(TIF)

S6 Fig The PKA inhibitor H89 promotes survival of BIPPO-treated but not cycle 1 PfPDEβ-null ring stages. (A) Representative Giemsa images of RAP- and mock-treated PfPDEβΔcatHA during cycle 1 post-excision show condensed forms appearing within a few hours after invasion. (B) RAP- and mock-treated PfPDEβΔcatHA were synchronised to a 2-hour invasion window and inhibitors added to the indicated concentrations at 0 to 2 hours post-invasion. Blood smears were taken at 20 to 22 hpi, stained with Giemsa, and imaged. (C) Giemsa-stained smears from ring stage cultures (18–22 hpi) treated with the indicated compounds. Kinase inhibitors (H89 and C2) were added at 1–5 hpi and BIPPO at 2–6 hpi. BIPPO, 5-Benzyl-3-isopropyl-1*H*-pyrazolo[4,3-*d*]pyrimidin-7(6*H*)-one; C2, Compound 2; hpi, hours post-invasion; PfPDEβ, *Plasmodium falciparum* phosphodiesterase β; PKA, cAMP-dependent protein kinase; RAP, rapamycin.
(TIF)

S1 Video (Related to Fig 4). Time-lapse video of egress in a mock-treated PfPDEβΔcatHA schizont. PfPDEβ, *Plasmodium falciparum* phosphodiesterase β.
(MOV)

S2 Video (Related to Fig 4). Time-lapse video of egress in a RAP-treated PfPDEβΔcatHA schizont. PfPDEβ, *Plasmodium falciparum* phosphodiesterase β; RAP, rapamycin.
(MOV)

S3 Video (Related to Fig 4). Time-lapse video of invasion in mock-treated PfPDEβΔcatHA merozoites (replicate 1). PfPDEβ, *Plasmodium falciparum* phosphodiesterase β.
(MOV)

S4 Video (Related to Fig 4). Time-lapse video of invasion in mock-treated PfPDEβΔcatHA merozoites (replicate 2). PfPDEβ, *Plasmodium falciparum* phosphodiesterase β.
(MOV)

S5 Video (Related to Fig 4). Time-lapse video of invasion in RAP-treated PfPDEβΔcatHA merozoites showing deformation of erythrocyte. PfPDEβ, *Plasmodium falciparum* phosphodiesterase β; RAP, rapamycin.
(MOV)

S6 Video (Related to Fig 4). Time-lapse video of invasion in RAP-treated PfPDEβΔcatHA merozoites showing deformation of erythrocyte and echinocytosis. PfPDEβ, *Plasmodium falciparum* phosphodiesterase β; RAP, rapamycin.
(MOV)

S7 Video (Related to Fig 4). Time-lapse video of invasion in RAP-treated PfPDEβΔcatHA merozoites showing deformation of erythrocyte, echinocytosis, and invasion. PfPDEβ, *Plasmodium falciparum* phosphodiesterase β; RAP, rapamycin.
(MOV)

S1 Table (Related to Fig 5). Quantitative mass spectrometric global phosphoproteome analysis data. Proteins with phosphosites up-regulated 2-fold are highlighted in darker green and those significantly up-regulated (Welch *t* test) in lighter green. Proteins with phosphosites down-regulated 2-fold are highlighted in darker pink and those significantly down-regulated (Welch *t* test) in lighter pink.
(XLSX)

S2 Table (Related to Fig 5). Protein levels as calculated from the quantification of unphosphorylated peptides present in the phosphopeptide-enriched sample. The two RAP-binding proteins fused to the two halves of the split Cre recombinase up-regulated >2-fold are highlighted in green. Significantly down-regulated proteins are highlighted in pink. RAP, rapamycin.
(XLSX)

S3 Table (Related to Fig 5). Rank-based (1D) annotation motif analysis revealing that PKA-like substrate motifs are the most highly enriched motifs found in phosphosites up-regulated in RAP-treated, compared with mock-treated, PfPDEβΔcatHA schizonts. Numbers of detected phosphosites for each motif are shown in the column ‘Size’, along with test score, *p*-values, and mean log2 fold changes. PfPDEβ, *Plasmodium falciparum* phosphodiesterase β; PKA, cAMP-dependent protein kinase; RAP, rapamycin.
(XLSX)

S4 Table (Related to Fig 5). Data underlying the GO analysis presented in Fig 5E. Proteins that make up the GO terms presented in the bar chart and individual phosphosites in these proteins are listed in sheet 1. Phosphosites displaying a PKA consensus motif are underlined. The individual numerical values underlying the bar chart are listed in sheet 2. GO, gene ontology; PKA, cAMP-dependent protein kinase.
(XLSX)

S1 Data Excel file containing the underlying numerical data for Fig 1D. (XLSX)

S2 Data Excel file containing the underlying numerical data for Fig 2E. (XLSX)

S3 Data Excel spreadsheet containing, in separate sheets, the underlying numerical data for Fig 3C and 3E. (XLSX)

S4 Data Excel spreadsheet containing, in separate sheets, the underlying numerical data for Fig 4A, 4B, 4C, 4D, 4E and 4E. (XLSX)

S5 Data Excel spreadsheet containing, in separate sheets, the underlying numerical data for Fig 5D and 5E. (XLSX)

S6 Data Excel spreadsheet containing, in separate sheets, the underlying numerical data for Fig 6E and 6G. (XLSX)

S7 Data Excel spreadsheet containing the underlying numerical data for Fig 7B. (XLSX)

S8 Data Excel spreadsheet containing the underlying numerical data for S3B Fig. (XLSX)

S9 Data Excel spreadsheet containing, in separate sheets, the underlying numerical data for S4A, S4B, S4C and S4D Fig. (XLSX)

## Abbreviations

3×HA: triple haemagglutinin
AMA1: apical membrane antigen-1
ATP: adenosine triphosphate
BAPTA-AM: 1,2-bis(*o*-aminophenoxy)ethane-*N*,*N*,*N′*,*N′*-tetraacetic acid-acetoxymethyl ester
BIPPO: 5-Benzyl-3-isopropyl-1*H*-pyrazolo[4,3-*d*]pyrimidin-7(6*H*)-one
cAMP: cyclic AMP
CDPK1: calcium-dependent protein kinase 1
cGMP: cyclic GMP
DiCre: dimerisable Cre recombinase
EBA175: erythrocyte-binding antigen 175
ER: endoplasmic reticulum
GO: gene ontology
GSK3: glycogen synthase kinase
GTP: guanosine triphosphate
HA: haemagglutinin
HCD: higher-energy collision dissociation
hDHFR: human dihydrofolate reductase
IFA: immunofluorescence assay
IMC: inner membrane complex
MSP1: merozoite surface protein 1
mTOR: mechanistic target of rapamycin
MyoA: myosin A
PDE: phosphodiesterase
PDEβ: phosphodiesterase β
PfACβ: *Plasmodium falciparum* adenylyl cyclase β
PfPDEβ: *Plasmodium falciparum* phosphodiesterase β
PKA: cAMP-dependent protein kinase
PKG: cGMP-dependent protein kinase
PV: parasitophorous vacuole
PVM: parasitophorous vacuole membrane
RAP: rapamycin
ROM4: rhomboid-like protease 4
SERA5: serine repeat antigen 5
SPA: scintillation proximity assay
SUB2: subtilisin-like protease 2
