# Supplementary figures and images for: Phosphodiesterase beta is the master regulator of cAMP signalling during malaria parasite invasion
Source: PLoS Biol. 2019 Feb 22;17(2):e3000154. doi: 10.1371/journal.pbio.3000154 (PMC6402698; doi:10.1371/journal.pbio.3000154)

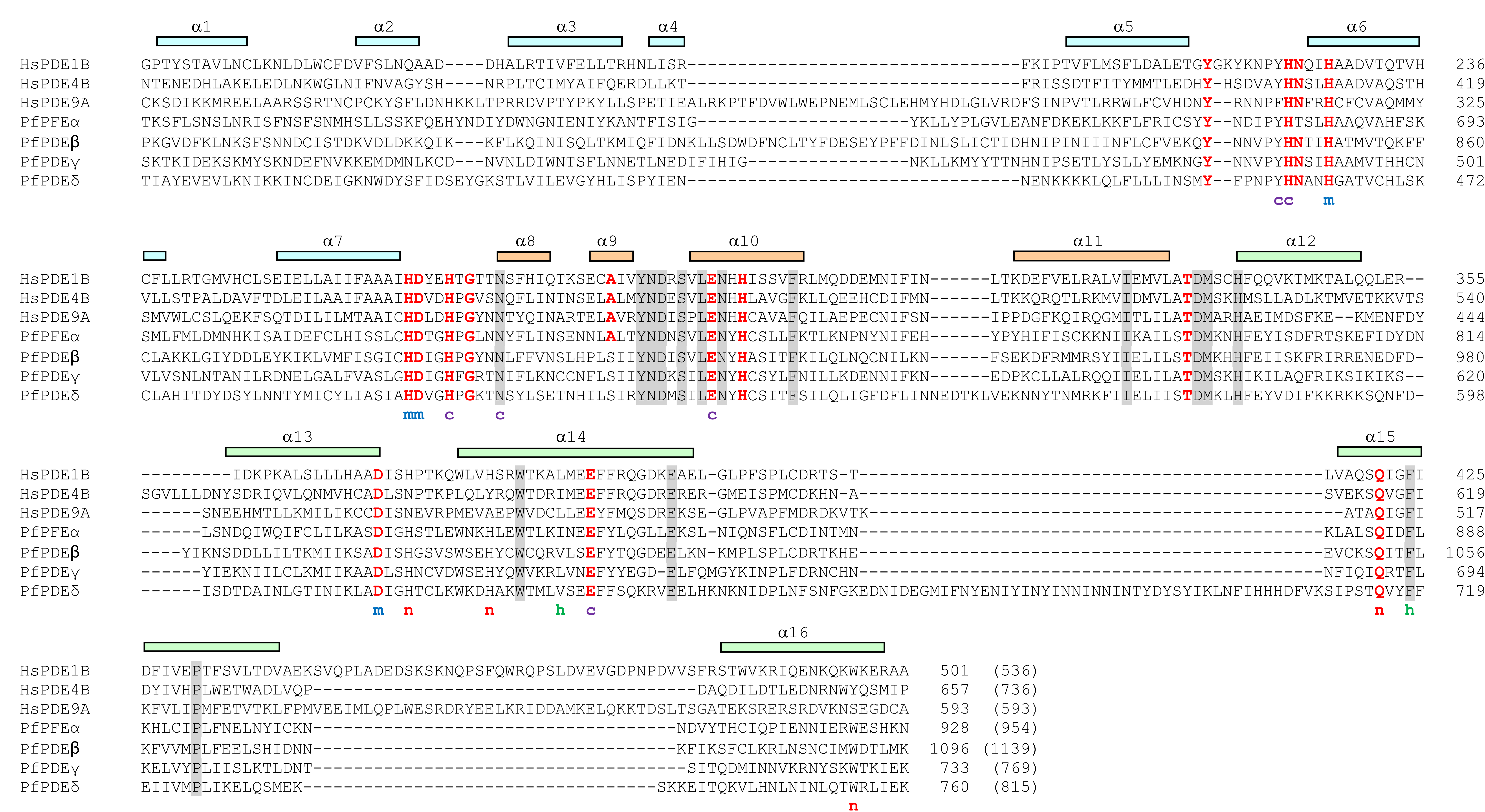

Supplement: S1 Fig — A total of 14 of the 15 residues (red) that are invariant amongst all of the 11 human PDE families are conserved in PfPDEβ. Additional residues identical in all the aligned sequences are highlighted in grey. Human PDE catalytic domains representing either dual-specific (HsPDE1, EAW96791), cAMP-specific (HsPDE4, NP_001032418), or cGMP-specific (HsPDE9A, AAC39778) types are aligned with the catalytic domains of the four P. falciparum PDEs. The 16 helical segments derived from studies on mammalian PDEs are depicted by coloured bars to reflect the three sub-domains: blue, NH2-terminal; orange, middle; and green, COOH-terminal, defined previously [58]. Functional residues defined in mammalian PDEs are indicated by the following: green ‘h’, hydrophobic clamp that binds the purine base; blue ‘m’, metal ion coordinating; red ‘n’, nucleotide recognition; and purple ‘c’, hydrolysis [59]. The amino acid numbers are shown to the right of the sequences, and the total number of amino acids in each protein is shown at the end in brackets. cAMP, cyclic AMP; cGMP, cyclic GMP; COOH, carboxyl; NH2, amino; PDE, phosphodiesterase; PfPDEβ, Plasmodium falciparum phosphodiesterase β. (TIF) [file pbio.3000154.s001.tif]

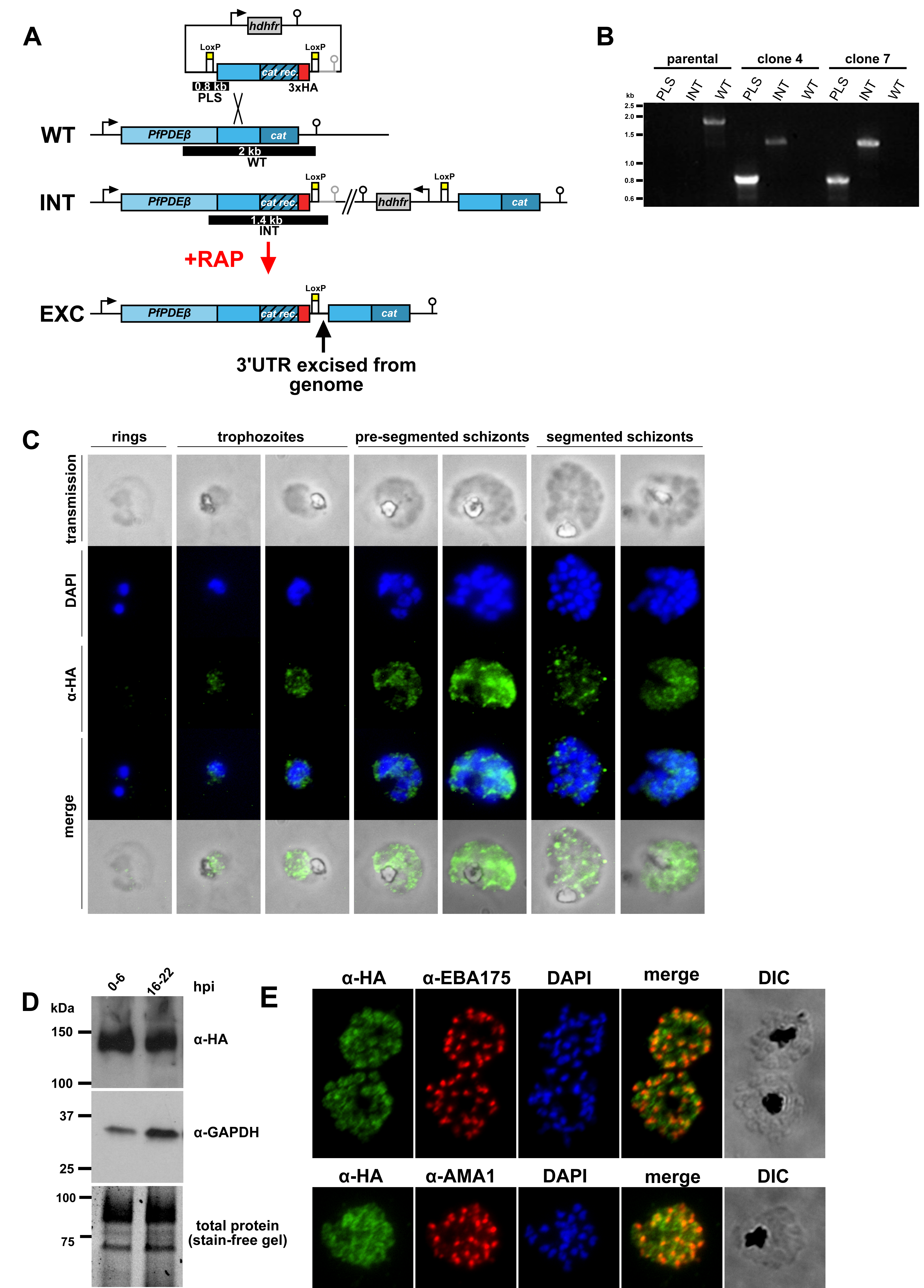

Supplement: S2 Fig — (A) Schematic showing the approach to C-terminally tag the endogenous PfPDEβ gene with a 3×HA tag. The plasmid construct was transfected into a line expressing a RAP-inducible Cre recombinase, upon activation of which the 3′ untranslated region (3′UTR) is excised. Excision of the 3′UTR did not result in the anticipated mRNA destabilisation and concomitant knockdown of protein levels. However, the created line proved useful for PDEβ localisation and enzymatic activity studies. Black arrows denote promoters and lollipops represent transcription terminators (grey circle represents the heterologous P. berghei dhfr-ts terminator). Positions of PCR amplicons verifying integration as well as absence of wild-type locus (see [B]) are indicated by black bars. (B) Diagnostic PCRs showing correct integration (INT) of the plasmid via single crossover into the PDEβ locus as well as absence of wild-type locus (WT) for two clones. The band obtained with primers specific for the plasmid (PLS) shows that multiple plasmid copies are integrated into the target locus. (C) Representative images of formaldehyde-fixed thin smears of ring, trophozoite, and schizont stages of PDEβ-HA parasites probed with rat anti-HA monoclonal antibody (green). Parasite nuclei are stained with DAPI (blue). (D) Full-length PfPDEβ-HA is expressed in early and late ring stages. Total lysates obtained from synchronous, high parasitaemia ring stage cultures were subjected to western blot analysis with monoclonal antibodies to the HA tag and the PfGAPDH. A section of the gel stained for total protein (stain-free gel) is shown as a loading control. (E) Dual-staining IFAs performed on thin smears of unblocked PfPDEβ-HA schizont cultures. Slides were stained with anti-HA (red), EBA175, or AMA1 (green). Nuclear material was visualised by DAPI (blue). Merged red and green channels are shown (merge) and a DIC microscopy image is shown to the right. Scale bar, 5 μm. AMA1, apical membrane antigen-1; DIC, differential int [file pbio.3000154.s002.tif]

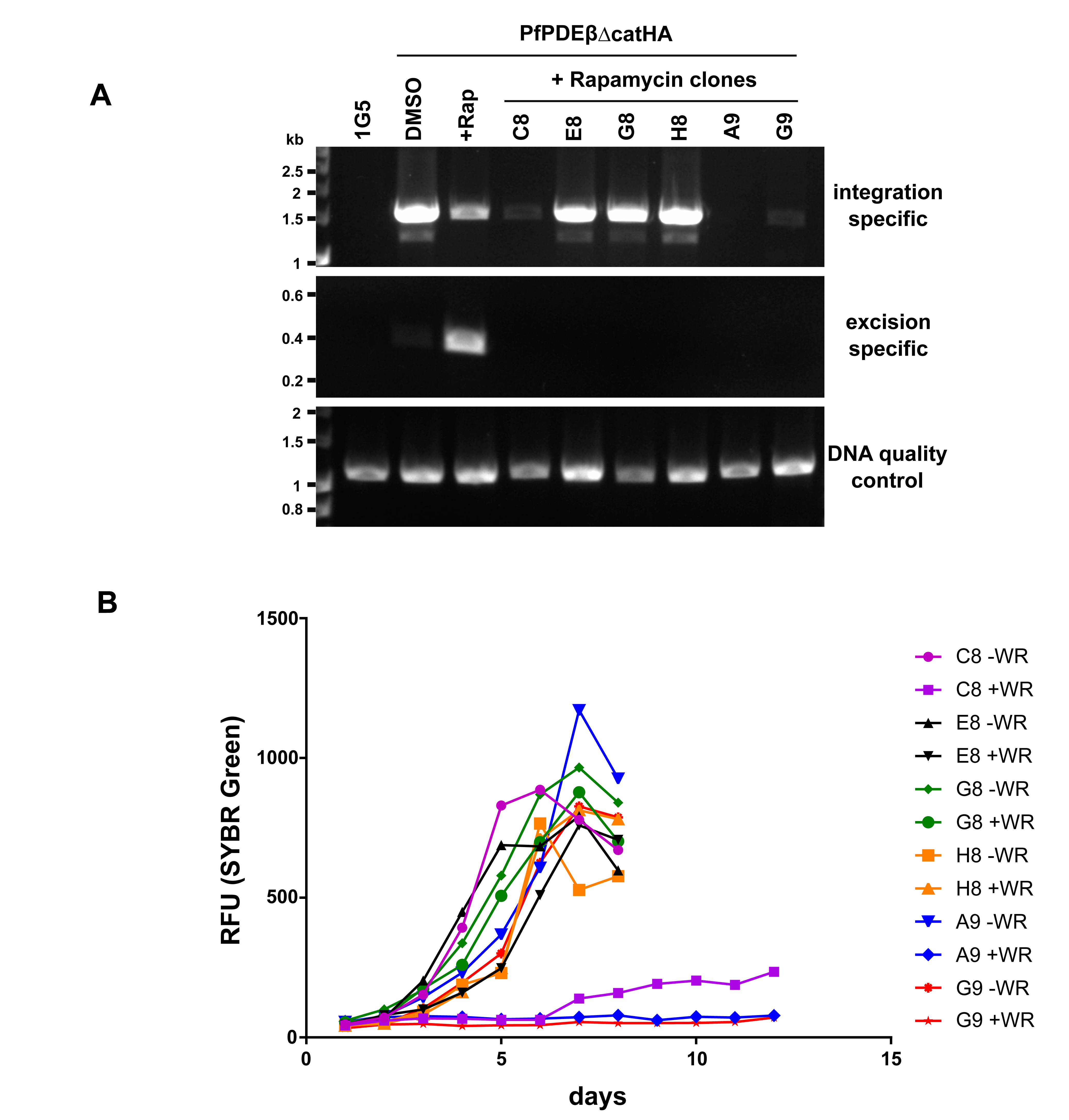

Supplement: S3 Fig — (A) PCR analysis of the PDEβ locus in six clones grown from a RAP-treated culture grown in the absence of WR99210 for 4 weeks. None of the six clones carried the excised PDEβ locus. A weak or absent integration-specific band is consistent with partial or full reversion of the unexcised PDEβ locus to wild type. (B) Growth curves for the six clones determined after 4 weeks of culture in the absence of WR99210 by daily measurements of DNA content via SYBR Green fluorescence (RFU). Means of technical triplicates are presented. Parasite clones were grown in the absence (−WR) and presence (+WR) of WR99210. Drug challenge reveals that three out of six clones had largely reverted to the drug-sensitive wild-type PDEβ locus. PDEβ, phosphodiesterase β; PfPDEβ, Plasmodium falciparum phosphodiesterase β; RAP, rapamycin; RFU, relative fluorescence unit; WR, WR99210. (TIF) [file pbio.3000154.s003.tif]

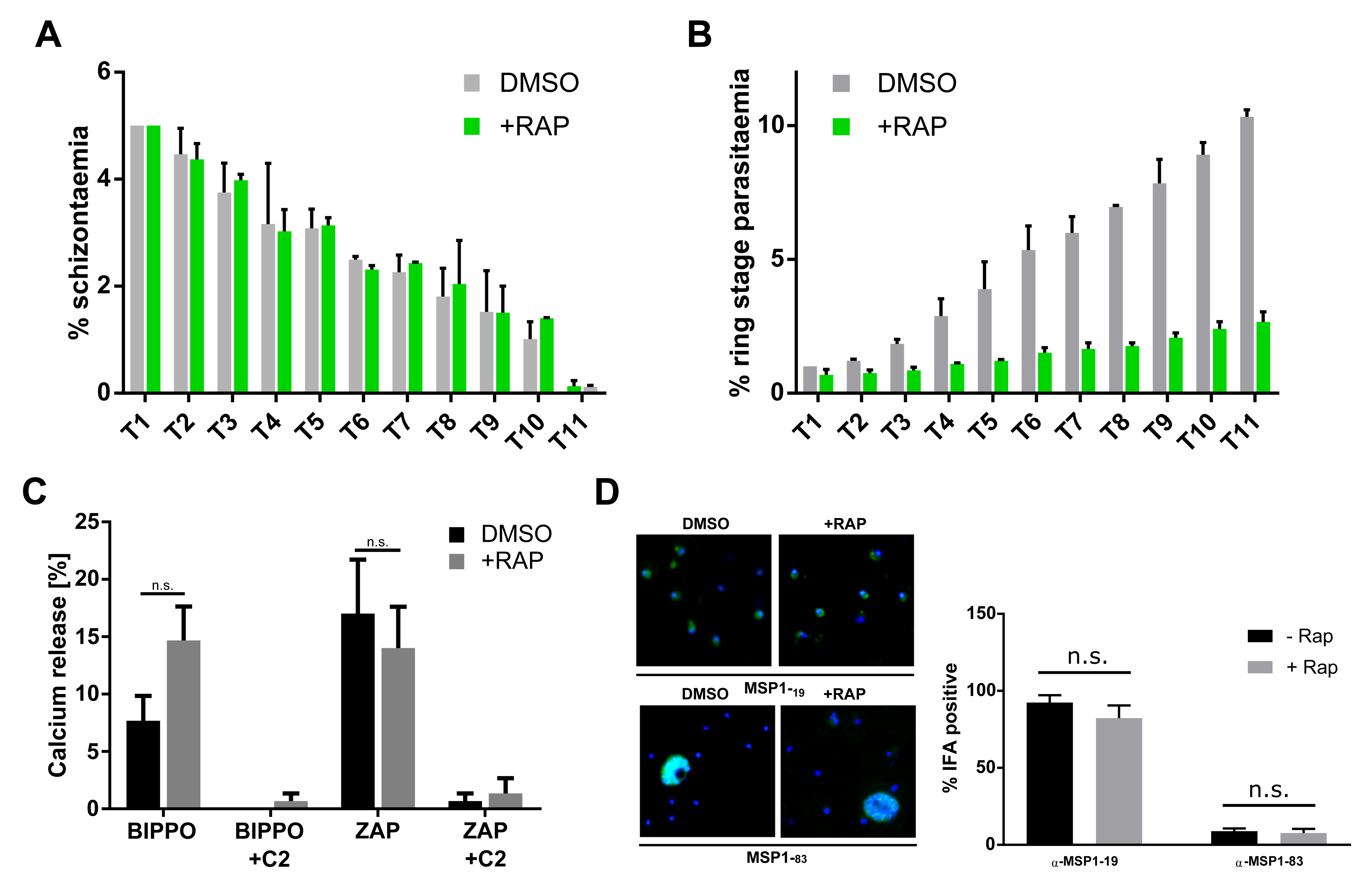

Supplement: S4 Fig — (A) Decline in schizontaemia over time in synchronous DMSO- and RAP-treated PfPDEβΔcatHA schizont cultures as determined by FACS on SYBR Green–stained cultures. Samples were taken every 45 minutes (T1–T10) and 12 hours later (T11). The data are mean schizontaemia (starting schizontaemia adjusted to 5%) from two biological replicates carried out in triplicate, and the error bars denote the standard deviation. (B) Increase in ring stage parasitaemia over time in synchronous DMSO- and RAP-treated PfPDEβΔcatHA cultures as determined by FACS on SYBR Green–stained cultures. Samples were taken every 45 minutes (T1–T10) and 12 hours later (T11). The data are mean ring stage parasitaemia (untreated starting parasitaemia adjusted to 1%) from two biological replicates carried out in triplicate, and the error bars denote the standard deviation. (C) DMSO- and +RAP–treated PfPDEβΔcatHA schizonts loaded with the membrane-permeable fluorescent calcium reporter Fluo-4 AM were exposed to PDE inhibitors BIPPO (2 μM) and zaprinast (ZAP) (100 μM), with or without the PKG inhibitor Compound 2 (C2) (2 μM), and calcium release measured as Fluo-4 fluorescence. Inhibitor-induced calcium release was normalised to values obtained for calcium ionophore, which was set to 100%. Data presented are means from three independent experiments performed in technical triplicates. Error bars, SEM. A paired t test showed no significant difference in response to either PDE inhibitor between wild-type (DMSO) and PDEβ KO schizonts. (D) IFA analysis suggests that mononucleated PDEβ knockout parasites are inside erythrocytes. Blood films of RAP- and mock-treated PfPDEβΔcatHA cultures containing segmented schizonts and early ring stages were stained with monoclonal antibodies recognising MSP1-83, which is shed at invasion, and MSP1-19, which is carried into the erythrocyte. There was no difference between the two conditions in the proportion of parasites staining with either antibody. Nonsignificant unpaired t t [file pbio.3000154.s004.tif]

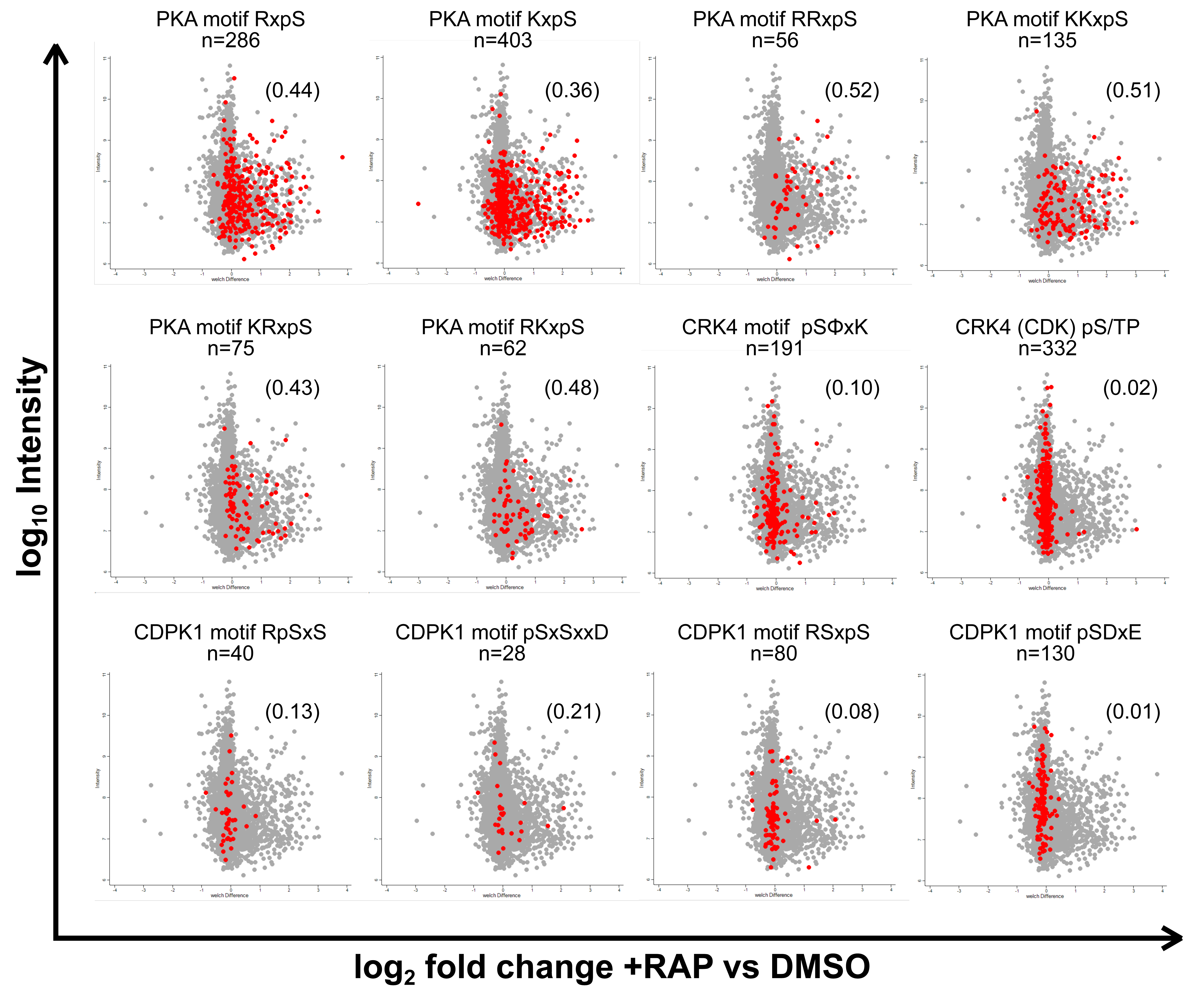

Supplement: S5 Fig — Intensity plots visualising log2 fold changes in phosphorylation at individual PKA and control motifs (in red) between PDEβ KO (+RAP) and wild-type (DMSO) schizonts. The total of 5,374 phosphosites are shown in grey. CRK4 motifs as identified by Ganter and colleagues, 2017 [56], and CDPK1 motifs as identified by Kumar and colleagues, 2017 [55], were unchanged. Motifs are shown above graphs. n = total number of phosphosites identified with a particular motif. Numbers in parentheses denote the ratio of phosphosites with a particular motif that were found to be significantly increased (Welch t test) in the PDEβ KO sample. CDPK1, calcium-dependent protein kinase 1; CRK4, cdc2-related protein kinase 4; KO, knockout; PDEβ, phosphodiesterase β; PKA, cAMP-dependent protein kinase; RAP, rapamycin. (TIF) [file pbio.3000154.s005.tif]

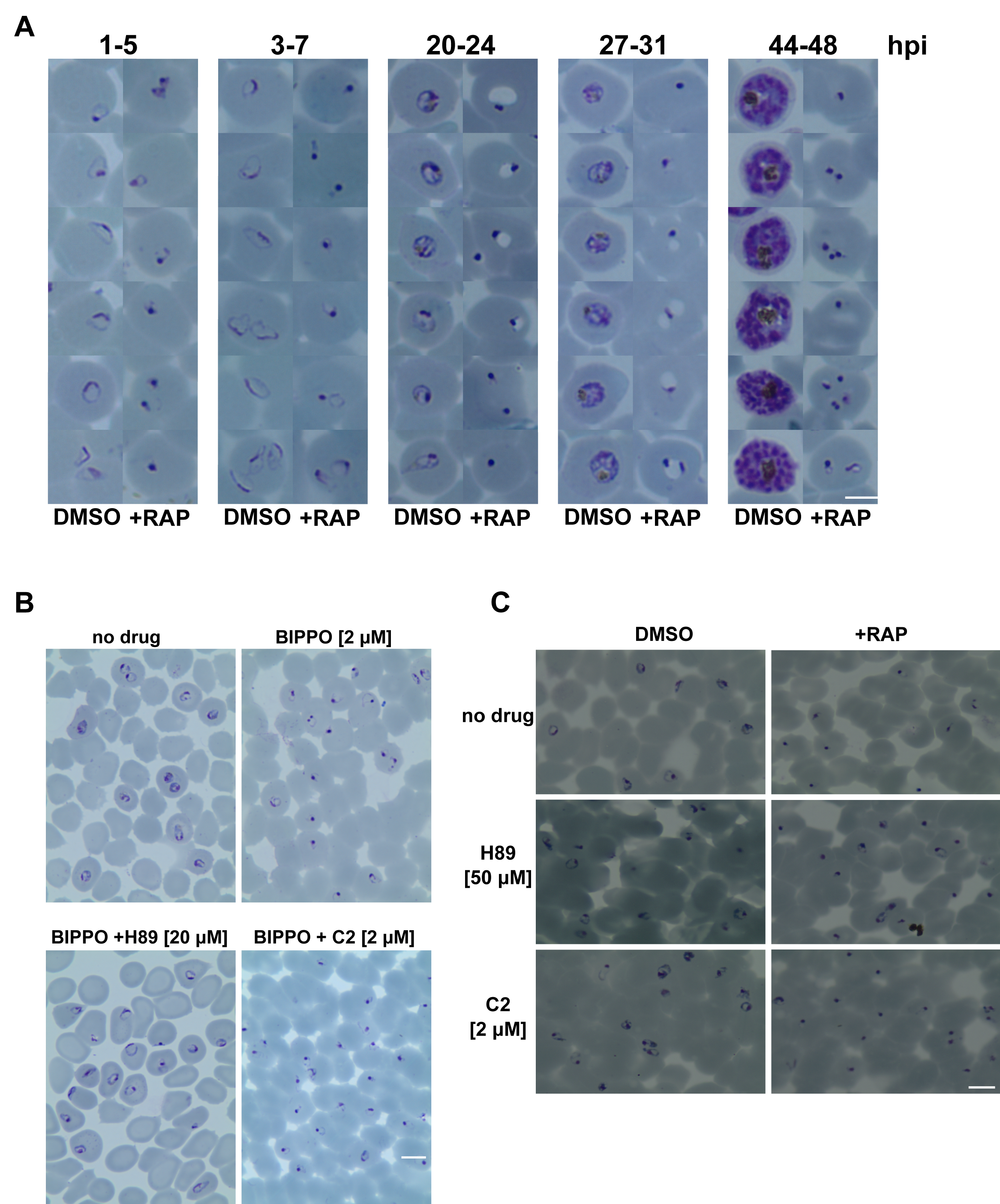

Supplement: S6 Fig — (A) Representative Giemsa images of RAP- and mock-treated PfPDEβΔcatHA during cycle 1 post-excision show condensed forms appearing within a few hours after invasion. (B) RAP- and mock-treated PfPDEβΔcatHA were synchronised to a 2-hour invasion window and inhibitors added to the indicated concentrations at 0 to 2 hours post-invasion. Blood smears were taken at 20 to 22 hpi, stained with Giemsa, and imaged. (C) Giemsa-stained smears from ring stage cultures (18–22 hpi) treated with the indicated compounds. Kinase inhibitors (H89 and C2) were added at 1–5 hpi and BIPPO at 2–6 hpi. BIPPO, 5-Benzyl-3-isopropyl-1H-pyrazolo[4,3-d]pyrimidin-7(6H)-one; C2, Compound 2; hpi, hours post-invasion; PfPDEβ, Plasmodium falciparum phosphodiesterase β; PKA, cAMP-dependent protein kinase; RAP, rapamycin. (TIF) [file pbio.3000154.s006.tif]
